# Anti-O-specific polysaccharide (OSP) immune responses following vaccination with oral cholera vaccine CVD 103-HgR correlate with protection against cholera after infection with wild-type *Vibrio cholerae* O1 El Tor Inaba in North American volunteers

**DOI:** 10.1371/journal.pntd.0006376

**Published:** 2018-04-06

**Authors:** Kamrul Islam, Motaher Hossain, Meagan Kelly, Leslie M. Mayo Smith, Richelle C. Charles, Taufiqur Rahman Bhuiyan, Pavol Kováč, Peng Xu, Regina C. LaRocque, Stephen B. Calderwood, Jakub K. Simon, Wilbur H. Chen, Douglas Haney, Michael Lock, Caroline E. Lyon, Beth D. Kirkpatrick, Mitchell Cohen, Myron M. Levine, Marc Gurwith, Jason B. Harris, Firdausi Qadri, Edward T. Ryan

**Affiliations:** 1 Division of Infectious Diseases, Massachusetts General Hospital, Boston, Massachusetts, United States of America; 2 Infectious Diseases Division, International Centre for Diarrhoeal Disease Research, Bangladesh (icddr,b), Dhaka, Bangladesh; 3 Harvard Medical School, Boston, Massachusetts, United States of America; 4 National Institute of Diabetes, Digestive and Kidney Diseases (NIDDK), Laboratory of Bioorganic Chemistry (LBC), National Institutes of Health, Bethesda, Maryland, United States of America; 5 Merck & Co., Inc., Kenilworth, New Jersey, United States of America; 6 Center for Vaccine Development, University of Maryland School of Medicine, Baltimore, Maryland, United States of America; 7 PaxVax, Inc., Redwood City, California, United States of America; 8 Vaccine Testing Center, Department of Medicine University of Vermont College of Medicine, Burlington, Vermont, United States of America; 9 Cincinnati Children’s Hospital Medical Center, and the Department of Pediatrics, University of Cincinnati College of Medicine, Cincinnati, Ohio, United States of America; 10 Department of Pediatrics, Harvard Medical School, Boston, Massachusetts, United States of America; 11 Department of Immunology and Infectious Disease, Harvard T.H. Chan School of Public Health, Boston, Massachusetts, United States of America; University of Tennessee, UNITED STATES

## Abstract

**Background:**

Cholera is an acute voluminous dehydrating diarrheal disease caused by toxigenic strains of *Vibrio cholerae* O1 and occasionally O139. A growing body of evidence indicates that immune responses targeting the O-specific polysaccharide (OSP) of *V*. *cholerae* are involved in mediating protection against cholera. We therefore assessed whether antibody responses against OSP occur after vaccination with live attenuated oral cholera vaccine CVD 103-HgR, and whether such responses correlate with protection against cholera.

**Methodology:**

We assessed adult North American volunteers (n = 46) who were vaccinated with 5 × 10^8^ colony-forming units (CFU) of oral cholera vaccine CVD 103-HgR and then orally challenged with approximately 1 × 10^5^ CFU of wild-type *V*. *cholerae* O1 El Tor Inaba strain N16961, either 10 or 90 days post-vaccination.

**Principal findings:**

Vaccination was associated with induction of significant serum IgM and IgA anti-OSP and vibriocidal antibody responses within 10 days of vaccination. There was significant correlation between anti-OSP and vibriocidal antibody responses. IgM and IgA anti-OSP responses on day 10 following vaccination were associated with lower post-challenge stool volume (r = −0.44, *P* = 0.002; r = −0.36, *P* = 0.01; respectively), and none of 27 vaccinees who developed a ≥1.5 fold increase in any antibody isotype targeting OSP on day 10 following vaccination compared to baseline developed moderate or severe cholera following experimental challenge, while 5 of 19 who did not develop such anti-OSP responses did (*P* = 0.01).

**Conclusion:**

Oral vaccination with live attenuated cholera vaccine CVD 103-HgR induces antibodies that target *V*. *cholerae* OSP, and these anti-OSP responses correlate with protection against diarrhea following experimental challenge with *V*. *cholerae* O1.

**Trial registration:**

ClinicalTrials.gov NCT01895855

## Author summary

Cholera is a severe watery diarrheal disease, caused by pathogenic strains of *V*. *cholerae*. Protective immunity against cholera is serogroup specific, and serogroup specificity is determined by the O-specific polysaccharide (OSP) of *V*. *cholerae* lipopolysaccharide (LPS). Despite this, no previous work has directly assessed correlation of OSP-immune responses and protection against cholera. In this study, we assessed adult North American volunteer’s antibody responses targeting OSP after vaccination with live attenuated oral cholera vaccine CVD 103-HgR, and we assessed correlation of protection against cholera with such antibody responses. Oral vaccination was associated with the induction of significant IgM and IgA responses against OSP, and these responses correlated with vibriocidal responses. There was significant negative correlation of OSP responses and post-challenge stool volume, and none of the volunteers who developed an anti-OSP antibody responses of any isotype of ≥1.5 fold developed moderate or severe cholera following experimental challenge. In summary, vaccination with live attenuated oral cholera vaccine CVD 103-HgR induces antibodies that target *V*. *cholerae* OSP, and these responses highly correlate with protection against cholera.

## Introduction

*Vibrio cholerae* can be characterized into over 200 serogroups [1]. *V*. *cholerae* serogroups O1 and O139 are the causes of epidemic cholera, a severe dehydrating illness of humans [2]. *V*. *cholerae* O1 and O139 are noninvasive intestinal pathogens that express cholera toxin within the intestine of infected humans. Cholera toxin (CT) is an ADP-ribosylating protein that binds to intestinal epithelial cells, resulting in increased intracellular cAMP, and subsequent chloride, sodium, and water excretion by intestinal epithelial cells, resulting in the secretory diarrhea characteristic of cholera [3, 4]. The mechanistic mediators of protection against cholera are poorly defined. Immune responses against CT do not mediate meaningful protective immunity against cholera [5–7]. Protection against cholera is serogroup specific, two major serogroups of *V*. *cholerae* O1 are Ogawa and Inaba and serospecificity is mediated by the O-specific polysaccharide (OSP) component of lipopolysaccharide (LPS) [6, 8]. Currently, the best correlate of protection against cholera is the vibriocidal antibody response [9, 10]. The vibriocidal response can largely be adsorbed away by incubating serum with purified *V*. *cholerae* OSP, suggesting that OSP is the prime antigenic target assessed by the vibriocidal assay [11].

A live attenuated oral cholera vaccine, CVD 103-HgR (Vaxchora, PaxVax, California) was recently FDA-approved in the United States. CVD 103-HgR is an engineered attenuated derivative of *V*. *cholerae* O1 classical Inaba strain 569B [9]. In North American volunteers, a single dose of CVD 103-HgR provided protection against moderate and severe diarrhea following subsequent experimental cholera infection with wild-type *V*. *cholerae* O1 organisms [10], although some vaccine recipients did develop moderate or severe diarrhea following experimental challenge. Protection was highly correlated with vibriocidal responses in vaccine recipients, especially the fold-increase in the vibriocidal titer following vaccination [12]. We were therefore interested in evaluating whether immune responses against *V*. *cholerae* O1 OSP occur after vaccination with CVD 103-HgR, and whether such responses to vaccination correlate with protection against cholera following subsequent experimental challenge with wild type *V*. *cholerae* O1 organisms.

## Materials and methods

### Ethics statement

This study involved analysis of anonymized samples from a previously reported clinical trial; this current use was approved by the Partners-Massachusetts General Hospital Institutional Review Board, Boston, Massachusetts. The serum samples were collected in a previously reported clinical trial (http://clinicaltrials.gov/show/NCT01895855) that was approved by the Institutional Review Boards of the University of Maryland, Baltimore; Cincinnati Children’s Hospital Medical Center, Ohio; and University of Vermont Burlington, Vermont. Written informed consent was obtained from participating healthy adults 18–45 years of age.

### Study design

The clinical study during which samples were collected has been previously described [10]. We limited analysis to vaccinated and challenged study enrollees with available blood samples for all key time points (n = 46). Briefly, North American volunteers were immunized orally with 5 × 10^8^ colony-forming units (CFU) of CVD 103-HgR, and then orally challenged on either day 10 (n = 26) or day 90 (n = 20) with 1 × 10^5^ CFU of wild type *V*. *cholerae* O1 El Tor Inaba N16961, as previously described [10]. Blood was collected pre-vaccination (day 0) from all participants. For volunteers in the 10 day challenge cohort, blood was also collected on post-vaccination days 7, 10 (10 days after vaccination, but before challenge), 20 (20 days after vaccination, 10 days after challenge), 38 (38 days after vaccination; 28 days after challenge), and 180 days after vaccination (180 days after vaccination; 170 days after challenge). For volunteers in the 90 day challenge cohort, blood was also collected on post-vaccination days 7, 10, 28, 90 (90 days after vaccination, but before challenge), 100 (100 days after vaccination, 10 days after challenge), 118 days after vaccination (28 days after challenge), and 180 days after vaccination (90 days after challenge).

### Definition of diarrhea

For analyses using diarrhea following challenge as an efficacy endpoint, diarrhea was defined as the passage of ≥2 loose stools (grade 3–5) over a 48-hour period ≥200 mL on volume or a single loose stool ≥300 mL, as previously described [10]. Moderate or severe diarrhea was defined as the passage of at least 3.0 L or 5.0 L of loose stool, respectively [10].

### Immunology

Stored serum specimens were tested to measure IgM, IgA and IgG immune responses against *V*. *cholerae* OSP purified from PIC018 (Inaba, El Tor) and PIC158 (Ogawa El Tor), as previously described [11, 13, 14]. Seroconversion for anti-OSP antibody responses was defined as ≥1.5-fold rise in ELISA units over baseline (described below). Vibriocidal responses were assessed to Inaba strain PIC018 and Ogawa strain PIC158, as previously described [15]. Vibriocidal seroconversion was defined as a ≥4-fold increase in reciprocal end-titer over the day 0 (pre-vaccination) value.

### Enzyme-linked immunosorbent assays (ELISAs) for the detection of OSP-specific IgM, IgA, and IgG antibodies responses in serum

We measured OSP-specific IgM, IgA and IgG responses in stored serum using standard enzyme-linked immunosorbent assay (ELISA) protocols, as described previously [15, 16]. In brief, to assess anti-OSP antibody responses, we coated ELISA plates (Nunc, Denmark) with OSPc:BSA (100 ng/well). To each well, we added 50 μL of serum (diluted 1:50 in 0.1% BSA in phosphate buffered saline-Tween) and then incubated plates for 90 minutes at 37°C. For detection of the presence of OSP-specific antibodies, we used horseradish peroxidase-conjugated anti-human IgM, IgA or IgG antibody (diluted 1:5000 in 0.1% BSA in phosphate buffered saline-Tween) (Jackson ImmunoResearch, USA). After 90 minutes incubation at 37°C, we developed plates with a 0.55 mg/mL solution of 2, 2ʹ-azinobis (3-ethylbenzothiazoline-6-sulfonic acid) (ABTS; Sigma) with 0.03% H_2_O_2_ (Sigma), and determined the optical density at 405 nm with a Vmax microplate kinetic reader (Molecular Devices Corp. Sunnyvale, CA). Plates were read for 5 min at 30s intervals, and the maximum slope for an optical density change of 0.2U was reported as millioptical density units per minute (mOD/min). We normalized ELISA units (EU) by calculating the ratio of the optical density of the test sample to that of a standard of convalescent-phase pooled sera (prepared from previously infected cholera patients) included on each plate.

### Statistical analyses

We analyzed differences in immune responses between groups using Mann-Whitney U tests. We used Fisher’s exact tests to assess differences by responder frequency. We used Spearman’s test to assess correlations of anti-OSP and vibriocidal antibody responses with cumulative diarrheal volume, as well as to assess correlations between immune responses. All *P* values were two-tailed, with a value of ≤0.05 considered the threshold for statistical significance. We performed analyses using GraphPad Prism, version 5.01 (GraphPad Software, Inc., La Jolla, CA).

## Results

### Study participants

Characteristics of study participants are described in Table 1.

**Table 1:** Characteristics of study participants.

Characteristics	Values (n = 46)	10 day challenge group (n = 26)	90 day challenge group (n = 20)
Gender (male) (%)	31 (67)	17 (65)	14 (70)
Median age in years (25^th^, 75^th^ centile)	31 (29, 38)	31 (28, 33)	33.5 (29, 43)
Blood group O (%)	24 (52)	11 (42)	13 (65)

### OSP-specific serum antibody responses following ingestion of oral cholera vaccine CVD 103-HgR

**Fig 1:**
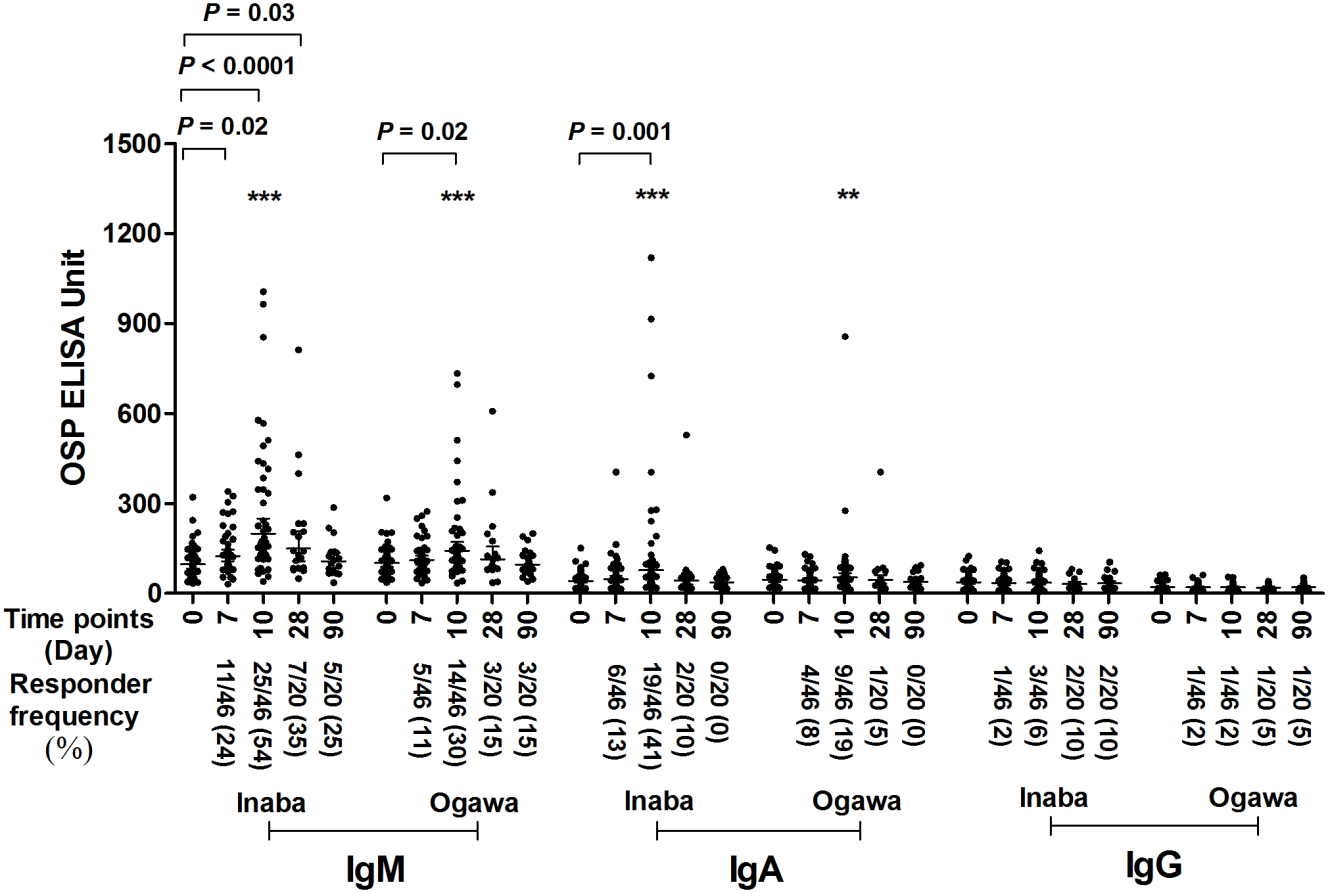
Serum IgM, IgA and IgG responses targeting *V*. *cholerae* O1 Inaba or Ogawa OSP in vaccine recipients of CVD 103-HgR. Figure only contains results for samples collected prior to wild type experimental *V*. *cholerae* challenge. Day 0 is pre-vaccination and other dates are timed from vaccination. In total, 46 vaccinees are included until day 10, then 20 vaccinees until day 90. X axis indicates the time points of samples while Y-axis denotes OSP-specific antibody responses. Each single dot indicates an individual OSP antibody value, horizontal bars indicate the geometric mean (GM), and error bars indicate 95% confidence intervals. *P* values represent significant differences of the mean between groups. Asterisks represent differences in responder frequency in Fisher’s exact test (*** *P* ≤ 0.001, ** *P* ≤ 0.01). We defined a responder as having a ≥1.5-fold increase in anti-OSP units after vaccination compared to pre-vaccination value. Responder frequency is represented in parenthesis on x-axis.

To assess anti-OSP responses following vaccination alone, we analyzed anti-OSP antibody responses in blood samples from both day 10 and day 90 challenge cohorts up to their respective days of challenge (Fig 1). We found a significant increase of the mean serum IgM response to *V*. *cholerae* O1 Inaba OSP within 7 days of oral vaccination with CVD 103-HgR, and these values remained elevated until at least day 28 following vaccination. The peak effect was seen at 10 days after vaccination. In the absence of wild type experimental challenge, anti-OSP Inaba IgM mean responses decreased to baseline by day 90 following vaccination. Elevated IgA anti-OSP Inaba mean increases were also present within 10 days following vaccination, and decreased to baseline by day 28. Anti-Ogawa IgM and IgA mean antibody increases were also induced following vaccination with CVD 103-HgR, although these responses were generally of lower magnitude and shorter duration than responses targeting Inaba OSP, consistent with the fact that CVD 103-HgR is an Inaba-based vaccine. Mean increases in anti-OSP IgG were not detected following vaccination.

### OSP-specific serum antibody responses following oral vaccination with CVD 103-HgR followed by experimental wild type oral challenge on day 10 or day 90 post-vaccination

**Fig 2:**
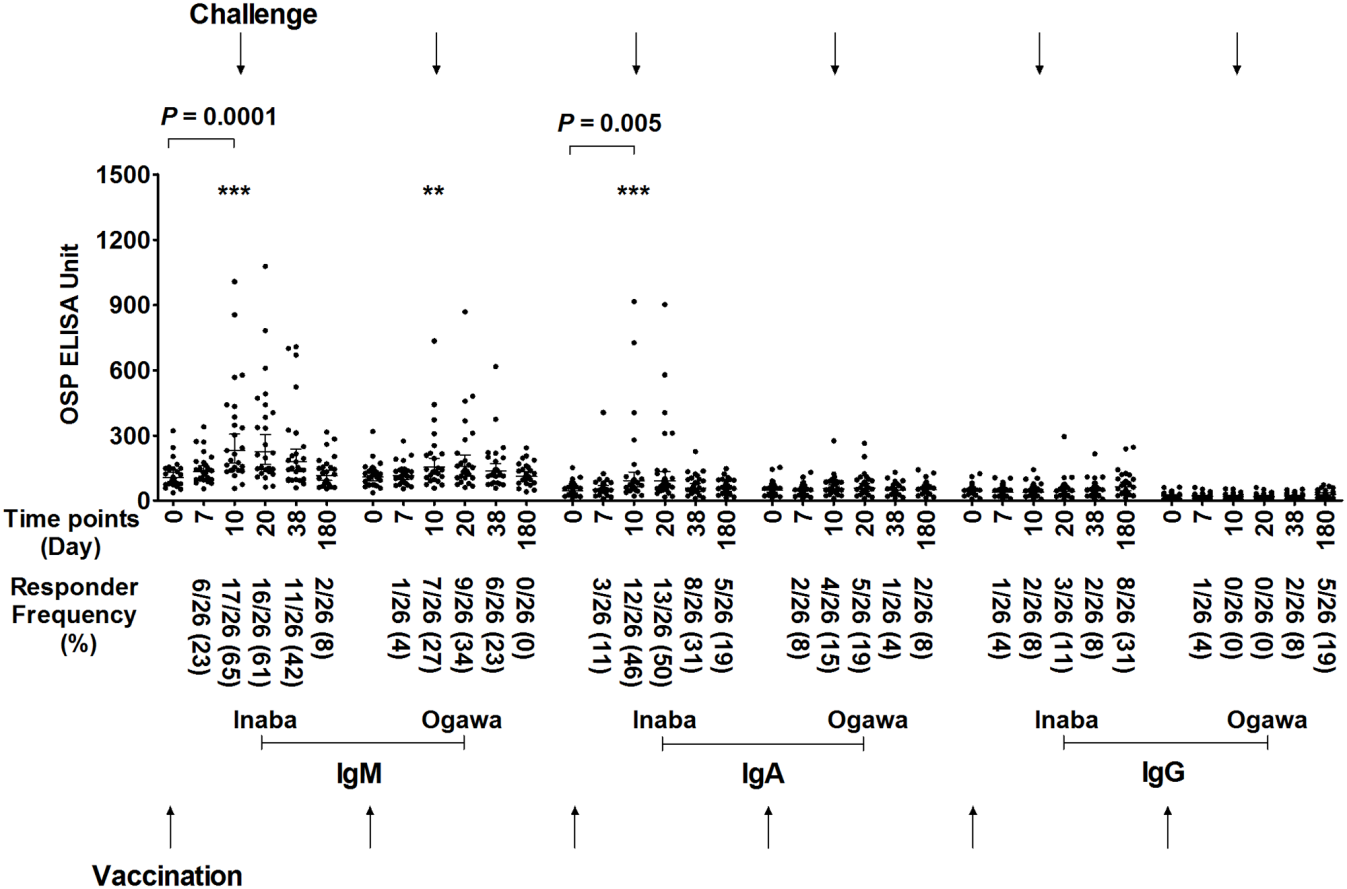
Serum antibody responses against OSP in vaccinees of day 10 challenged group. Serum IgM, IgA and IgG responses targeting *V*. *cholerae* O1 Inaba or Ogawa OSP in recipients (n = 26) of oral cholera vaccine CVD 103-HgR who were then experimentally challenged with wild type *V*. *cholerae* O1 Inaba N16961 10 days after vaccination (denoted by top arrows). Day 0 is pre-vaccination and other dates are timed from vaccination. X axis indicates the time points of samples, while Y-axis denotes OSP-specific antibody responses. Each single dot indicates an individual OSP antibody value, horizontal bars indicate the geometric mean (GM), and error bars indicate 95% confidence intervals. *P* values represent differences of the mean between groups. Asterisks represent significant differences in responder frequency in Fisher’s exact test (*** *P* ≤ 0.001, ** *P* ≤ 0.01). We defined a responder as having a ≥1.5-fold increase in anti-OSP units compared to pre-vaccination value. Responder frequency is represented in parenthesis on x-axis.

**Fig 3:**
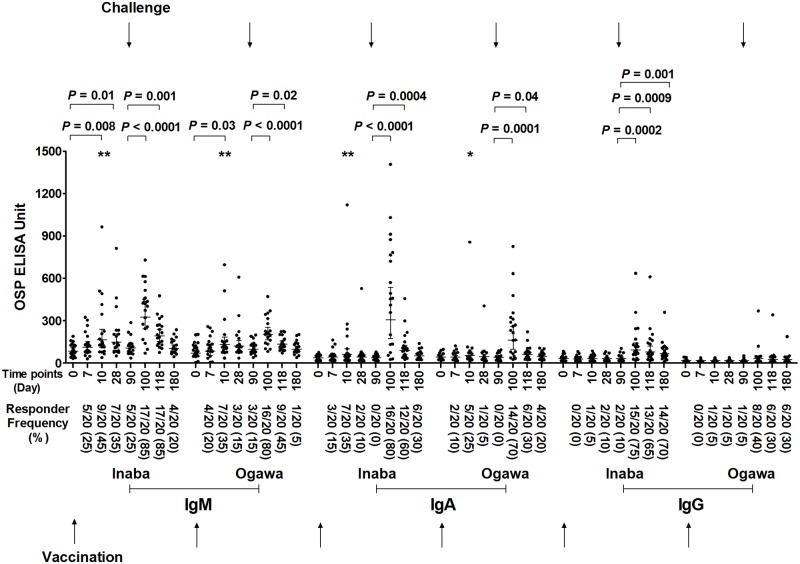
Serum antibody responses against OSP in vaccinees of day 90 challenged group. Serum IgM, IgA and IgG responses targeting *V*. *cholerae* O1 Inaba or Ogawa OSP in recipients (n = 20) of oral cholera vaccine CVD 103-HgR who were then experimentally challenged with wild type *V*. *cholerae* O1 Inaba N16961 90 days after vaccination (denoted by top arrows). Day 0 is pre-vaccination and other dates are timed from vaccination. X axis indicates the time points of samples, while Y-axis denotes OSP-specific antibody responses. Each single dot indicates an individual OSP antibody value, horizontal bars indicate the geometric mean (GM), and error bars indicate 95% confidence intervals. *P* values represent differences of the mean between groups. Asterisks represent significant differences of responder frequency in Fisher’s exact test (*** *P ≤* 0.001, ** *P ≤* 0.01). We defined a responder as having a ≥1.5-fold increase in anti-OSP units compared to pre-vaccination value. Responder frequency is represented in parenthesis on x-axis.

We found no significant boosting of IgM or IgA anti-OSP mean responses following challenge, when analyzing immune responses in the cohort of volunteers vaccinated with CVD 103-HgR challenged 10 days after vaccination (Fig 2). In comparison, we found significant boosting of anti-Inaba OSP and anti-Ogawa OSP immune responses of both IgM and IgA isotypes in volunteers vaccinated with CVD 103-HgR who were then challenged 90 days after vaccination (Fig 3). We also detected an increase in mean anti-OSP IgG responses following challenge in the cohort of vaccinees who were challenged 90 days post-vaccination.

### Immune responses to OSP in blood group O and non-O volunteers immunized with CVD 103-HgR

We did not detect a difference in mean antibody responses targeting OSP following vaccination when considering cohorts by blood group O characterization (S1 Fig).

### Immune responses targeting OSP 10 days after vaccination with CVD 103-HgR are associated with protection against experimental challenge with wild type *V*. *cholerae* O1

**Fig 4:**
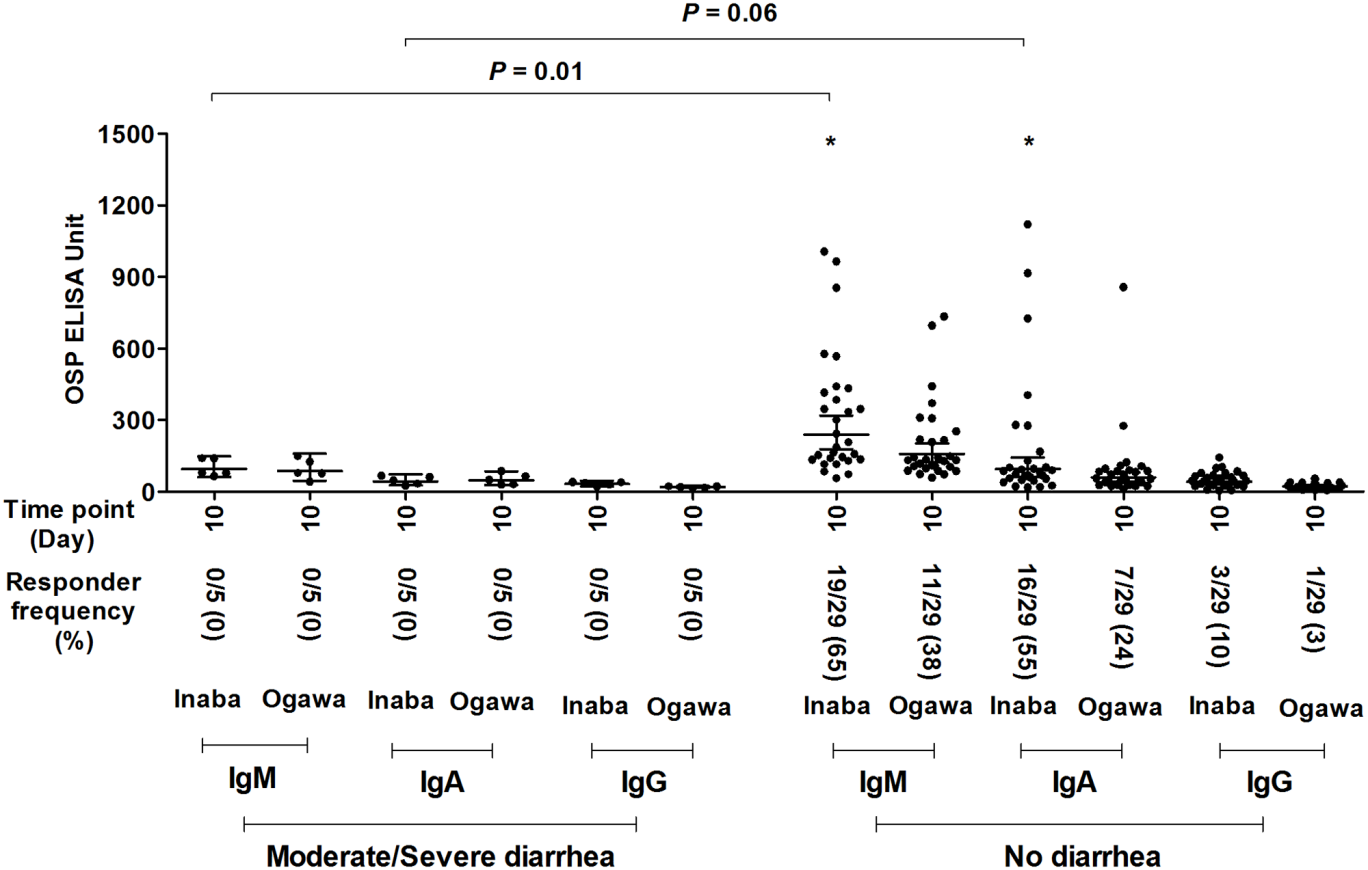
Serum IgM, IgA and IgG antibody responses targeting *V*. *cholerae* O1 Inaba and Ogawa OSP 10 days after oral vaccination. The figure shows the OSP specific antibodies responses 10 days after vaccination in volunteers who developed moderate (≥3L) or severe (≥5L) diarrhea versus no diarrhea following wild type *V*. *cholerae* O1 Inaba experimental infection 10 or 90 days after vaccination. X axis indicates the time points of samples while Y-axis denotes OSP specific antibody responses. Each single dot indicates an individual OSP antibody value, horizontal bars indicate the geometric mean (GM), and error bars indicate 95% confidence intervals. Responders were defined as having an increase in kinetic ELISA units ≥ 1.5 fold on day 10 post-vaccination compared with day 0 pre-vaccination. Asterisk indicates statistical significant difference (* *P* ≤ 0.05) of responder frequency between no diarrhea versus moderate/severe diarrhea group in Fisher’s exact test.

In our analyzed cohort of 46 volunteers, vaccination with CVD 103-HgR was associated with 92% protection against moderate or severe diarrhea following experimental challenge 10 days after vaccination, and 85% protection against moderate or severe diarrhea following 90 day challenge (Table 2). Due to the low number of vaccinated volunteers who developed moderate or severe diarrhea following challenge with wild type *V*. *cholerae* (2 of 26 in 10 day challenge cohort, and 3 of 20 in the 90 day cohort), for the purposes of assessing whether anti-OSP responses correlated with protection against cholera, we combined day 10 and day 90 challenge cohorts, and assessed outcome by post-vaccination day 10 immune responses. We found that IgM anti-OSP Inaba responses on day 10 following vaccination with CVD 103-HgR correlated with protection against moderate or severe diarrhea following subsequent experimental challenge (moderate or severe diarrhea versus no diarrhea; *P* = 0.01; Fig 4), and that day 10 IgA anti-OSP responses approached significance in this analysis (*P* = 0.06; Fig 4). We found similar results when considering day 10 anti-Inaba OSP responses and protection from any degree of diarrhea, with differences in mean values approaching significance for both IgM (*P* = 0.06) and IgA (*P* = 0.05) (S2 Fig). Interestingly, although oral vaccination with CVD 103-HgR was a poor inducer of anti-OSP IgG responses, low level responses were induced in some vaccine recipients (3 of 46 vaccinees developed a ≥1.5 fold increase in anti-OSP IgG on day 10 compared to baseline values), and day 10 IgG mean responses were significantly associated with protection from any degree of diarrhea following experimental challenge (*P* = 0.01; S2 Fig). Anti-OSP IgM responses 10 days after vaccination were also associated with protection when comparing volunteers who developed moderate or severe diarrhea versus no-or-mild-diarrhea (*P* = 0.01; S3 Fig).

**Table 2:** Protection afforded by oral cholera vaccine CVD 103-HgR against diarrhea following subsequent experimental challenge [10].

Degree of diarrhea	Vaccinee 10 day challenge (n = 26)	Percentage (%)	Vaccinee 90 day challenge (n = 20)	Percentage (%)
No diarrhea	21	80.8	8	40
Mild	3	11.5	9	45
Moderate	1	3.9	1	5
Severe	1	3.9	2	10
Protection from moderate or severe diarrhea	92%		85%	

For evaluating diarrhea as an efficacy endpoint following challenge, diarrhea was defined as the passage of ≥2 loose stools over a 48-hour period of ≥200 mL or a single loose stool of ≥300 mL [10]. Moderate or severe diarrhea was defined as the passage of at least 3.0 L or 5.0 L of loose stool, respectively [10]. Protection was ascertained for the cohort used in this current study using previously reported data compared to placebo recipients [10].

### Correlation of fold-increase of OSP responses 10 days after vaccination compared to pre-vaccination and diarrheal amount following experimental infection

**Fig 5:**
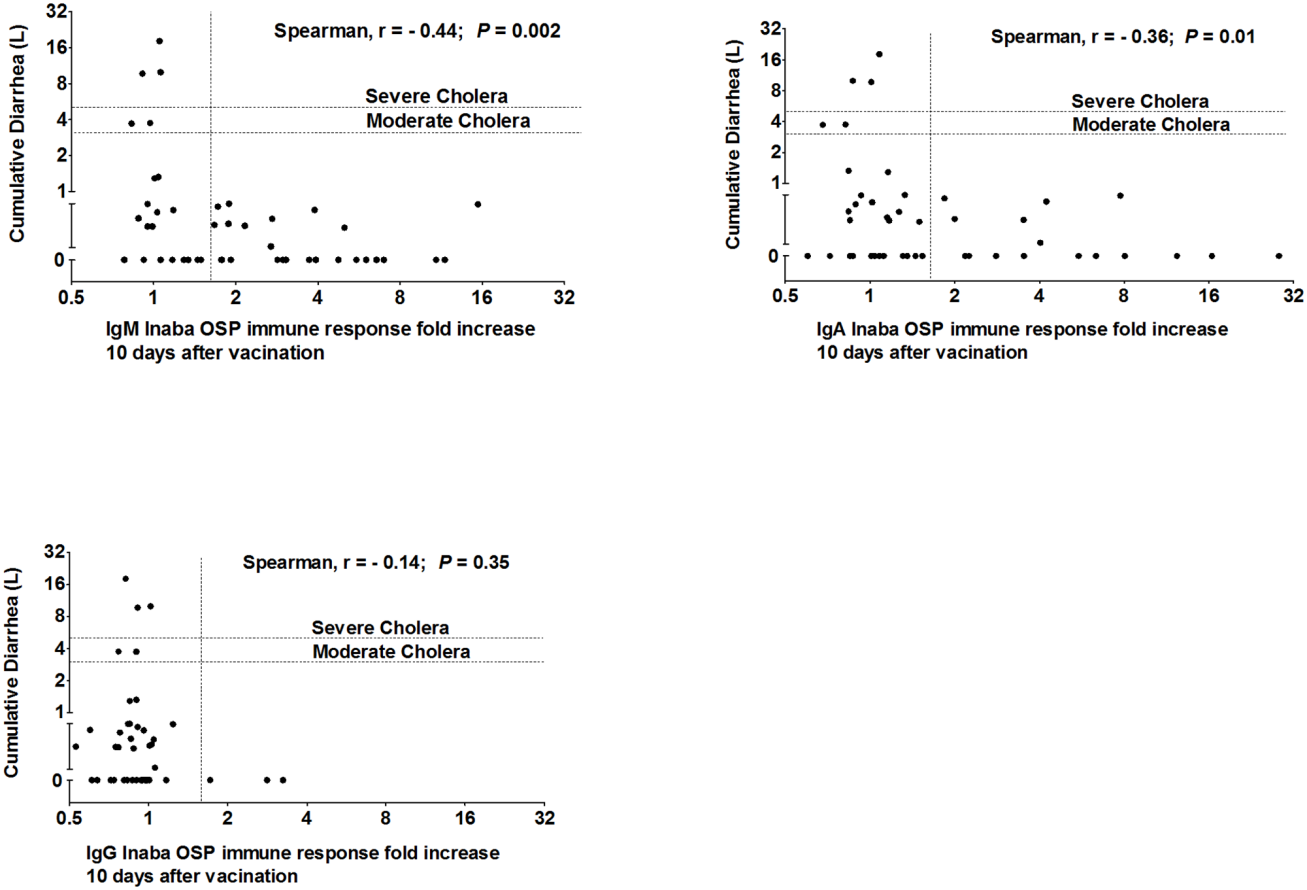
Correlation between fold increase of Inaba OSP specific antibody responses and cumulative diarrheal volume. X axis represents the fold-increase (day 10 to day 0) of IgM, IgA, and IgG responses against *V*. *cholerae* O1 Inaba OSP and Y axis represents cumulative diarrhea following experimental challenge with wild type *V*. *cholerae* O1 Inaba 10 or 90 days post vaccination with Inaba CVD 103-HgR. Dashed horizontal lines mark moderate (3L) or severe (5L) diarrheal values. Dashed vertical line denotes 1.5-fold anti-OSP antibody value change (day 10 post-vaccination compared to day 0 pre-vaccination).

We found significant negative correlation of the cumulative volume of diarrhea following experimental wild type *V*. *cholerae* O1 challenge and post-vaccination day 10 anti-Inaba OSP IgM responses (Spearman r = −0.44, *P* = 0.002) and anti-Inaba OSP IgA responses (r = −0.36, *P* = 0.01) (Fig 5), but no correlation with day 10 post-vaccination IgG responses. We found that a ≥1.5 fold increase in anti-OSP antibody responses highly correlated with protection against cholera: none of 27 vaccinees who developed ≥1.5 fold increase in any antibody isotype (IgM, IgA, IgG) targeting OSP on day 10 following vaccination compared to baseline developed moderate or severe cholera following experimental challenge, while 5 of 19 (26%) who did not develop any such anti-OSP responses did develop moderate or severe diarrhea (*P* = 0.01; Table 3 and S1 Table). For comparison, 39 of 66 placebo (59%) recipients who were part of the initial vaccine study from which the current vaccinee samples were analyzed developed moderate or severe diarrhea following experimental challenge [10]. In our current analysis, none of the 26 vaccinees who developed a ≥1.5 fold increase in IgM anti-OSP, none of 19 who developed a ≥1.5 anti-OSP IgA fold increase, and none of 3 who developed a ≥1.5 anti-OSP IgG fold increase on day 10 following vaccination compared to pre-vaccination values developed moderate or severe cholera following experimental challenge. In contrast, 5 of 19 (IgM; *P* = 0.01), 5 of 22 (IgA; *P* = 0.07), and 5 of 38 (IgG; *P* = 1.00), who did not develop such anti-OSP responses did develop moderate or severe diarrhea. Fold increase of anti-OSP values was also associated with protection against all degrees of diarrhea (mild, moderate, or severe) following wild type challenge. Six of 27 vaccinees who developed a ≥1.5 fold increase in any antibody isotype (IgM, IgA, IgG) targeting OSP on day 10 following vaccination compared to baseline developed mild diarrhea; none developed moderate or severe diarrhea following experimental challenge, while 11 of 19 who did not develop such anti-OSP responses did develop diarrhea (*P* = 0.03; Table 4 and S2 Table).

**Table 3:** Serum Inaba OSP-specific antibody isotype fold increase (≥1.5) and Inaba-specific vibriocidal fold increase (≥4) on day 10 following vaccination as a predictor of protection against development of moderate or severe diarrhea following subsequent challenge.

Inaba OSP specific antibody isotype fold increase; and Inaba vibriocidal fold increase	Fold increase on day10 *	Moderate or Severe diarrhea		*P* Value
		Yes	No	
Any of 3 isotypes or all IgM, A, G ≥1.5	Yes	0	27	0.01
	No	5	14	
Either or both IgM, A ≥1.5	Yes	0	27	0.01
	No	5	14	
Either or both IgM, G ≥1.5	Yes	0	26	0.01
	No	5	15	
Either or both IgA, G ≥1.5	Yes	0	19	0.07
	No	5	22	
IgM ≥1.5	Yes	0	26	0.01
	No	5	15	
IgA ≥1.5	Yes	0	19	0.07
	No	5	22	
IgG ≥1.5	Yes	0	3	1.00
	No	5	38	
Vibriocidal ≥ 4.0	Yes	2	39	0.01
	No	3	2	

*Represents day 10 fold increase after vaccination from day 0 anti-OSP or vibriocidal value; combining day 10 and day 90 challenge groups; *P* ≤ 0.05 considered significant in Fisher exact test

**Table 4:** Serum Inaba OSP-specific antibody isotype fold increase (≥1.5) and Inaba-specific vibriocidal fold increase (≥4) on day 10 following vaccination as a predictor of protection against development of mild, moderate or severe diarrhea following subsequent challenge.

Inaba OSP specific antibody isotype fold increase and Inaba vibriocidal fold increase	Fold increase on day10 *	Mild/ Moderate /Severe diarrhea		*P* Value
		Yes	No	
Any of 3 isotypes or all IgM, A, G ≥1.5	Yes	6	21	0.03
	No	11	8	
Either or both IgM, A ≥1.5	Yes	6	21	0.03
	No	11	8	
Either or both IgM, G ≥1.5	Yes	6	20	0.04
	No	11	9	
Either or both IgA, G ≥1.5	Yes	3	16	0.02
	No	14	13	
IgM ≥1.5	Yes	6	20	0.04
	No	11	9	
IgA ≥1.5	Yes	3	16	0.02
	No	14	13	
IgG ≥1.5	Yes	0	3	0.29
	No	17	26	
Vibriocidal ≥ 4.0	Yes	13	28	0.06
	No	4	1	

*Represents day 10 fold increase after vaccination from day 0 anti-OSP or vibriocidal value; combining day 10 and day 90 challenge groups

### Correlation of anti-OSP responses and vibriocidal responses

Vaccination with CVD 103-HgR induced prominent vibriocidal antibody responses (S4 Fig). These responses were present within 7 days of vaccination, peaked by day 10 following vaccination, and although falling toward baseline, mean values remained elevated over baseline on the last day evaluated (day 90). Anti-Inaba vibriocidal responses were more prominent than anti-Ogawa vibriocidal responses, and responses did not differ by blood group O status (S5 Fig). Similar to what was seen when analyzing anti-OSP responses, there was no boosting of vibriocidal responses when vaccinees were challenged 10 days after vaccination (S6 Fig), but there was significant boosting of vibriocidal responses when challenge was delayed to 90 days after vaccination (S7 Fig) for both Ogawa and Inaba. Vibriocidal responses 10 days after vaccination were associated with protection against subsequent challenge (S8–S10 Figs), as also previously reported [10]. We also found that a ≥4-fold increase of vibriocidal titer 10 days after vaccination was associated with protection against diarrhea following subsequent challenge (*P* = 0.01) (S11 and S12 Figs). Serum IgM OSP responses were most highly correlated with vibriocidal responses (Spearman r = 0.67; *P* < 0.0001) (S13 Fig). Serum IgA OSP responses correlated less well with vibriocidal responses (Spearman r = 0.32; *P* = 0.03), and there was no correlation of IgG OSP responses and vibriocidal responses (S13 Fig).

## Discussion

In this study, we found that recipients of live attenuated oral cholera vaccine CVD 103-HgR develop prominent immune responses targeting *V*. *cholerae* O-specific polysaccharide (OSP), and that these responses correlate with protection against moderate or severe diarrhea following experimental challenge with wild type *V*. *cholerae* O1. Antibodies targeting OSP induced by vaccination with CVD 103-HgR were largely of the IgM and IgA isotypes. Since *V*. *cholerae* is a noninvasive mucosal pathogen of the human intestine, IgM and IgA immune responses would be predicted to be the most pertinent isotypes involved in mechanistically mediating protection against cholera, since both IgM and IgA are actively secreted into the intestinal lumen by intestinal epithelial cells [17–19].

Induction of IgA antibody is associated with oral and mucosal vaccination, especially in response to protein antigens that are antigenically processed by T cells [20, 21]. OSP is a polysaccharide antigen, and as such, its antigenic processing would be predicted to be largely T cell independent [20, 21]. T cell independent immune responses to polysaccharide antigens usually result in predominantly IgM responses, since antibody maturation and isotype switching is not usually facilitated in the absence of T cell assistance [20, 21]. Antibody affinity in such situations is usually low, although antibody avidity can be prominent due to the pentameric nature of intra-luminal intestinal IgM, and the dimeric nature of intra-luminal intestinal IgA [21, 22]. Effector immune responses at mucosal surfaces are usually relatively short-lived [23, 24], although induction of long-lived plasma cells or memory B cells in intestinal tissue can facilitate rapid anamnestic responses [23, 24]. In our study, serum anti-OSP antibody responses fell back toward baseline within 90 days of vaccination, although possible persistence of antibody responses in mucosal tissue was not assayed in this study. Whether CVD 103-HgR induces long-lived plasma cell responses or memory B cell responses targeting *V*. *cholerae* OSP is currently unknown, as is the duration of protection afforded by vaccination.

In our study, IgM and, less so, IgA antibody responses targeting *V*. *cholerae* OSP highly correlated with vibriocidal responses in vaccine recipients. This would be expected, since we have previously shown that the vibriocidal response can largely be adsorbed away by *V*. *cholerae* OSP [11]. It is most likely that the vibriocidal response is largely a surrogate marker of as yet poorly defined mucosal immune responses targeting *V*. *cholerae*. The vibriocidal assay assesses complement-dependent mediated bacterial cell lysis in an *in vitro* assay [25, 26]. Bacterial lysis in the vibriocidal assay is cell-independent, and occurs via the terminal attack complex of complement [27, 28]. Mucosal IgA does not activate complement in the classical pathway, and in the setting of an intact intestinal epithelial border, it is thought that the components of the terminal attack complex of complement are not present in appreciable quantities in the intestinal lumen [27–29]. Our analysis showing high correlation of OSP antibody levels and the vibriocidal response may suggest that protection against cholera may actually be afforded at least in part by immune responses targeting *V*. *cholerae* OSP. Indeed, in our current analysis, we show that immune responses targeting *V*. *cholerae* OSP correlate with protection against experimental challenge with wild-type *V*. *cholerae*. This association was most clear when considering IgM responses targeting *V*. *cholerae* OSP, but also approached statistical significance (*P* = 0.06) when considering IgA responses targeting *V*. *cholerae* OSP. There was no association of mean IgG anti-OSP responses and protection against cholera. IgG is not actively transported into the intestinal lumen in appreciable quantities in the presence of an intact epithelium, and IgG responses would not be predicted to play a major role in mechanistically mediating protection against *V*. *cholerae*. The few vaccine recipients who did develop IgG responses targeting OSP in this study, also developed IgM and IgA anti-OSP responses.

Interestingly, we found no boosting of immune responses targeting *V*. *cholerae* OSP or vibriocidal responses when vaccine recipients were experimentally challenged with wild-type *V*. *cholerae* just 10 days after vaccination. We did, however, find significant boosting of anti-*V*. *cholerae* immune responses and vibriocidal responses following experimental challenge with wild-type *V*. *cholerae* when 90 days had elapsed since vaccination. A possible explanation for this observation is that mucosal immune responses 10 days following vaccination may have been so prominent that there was minimal if any immunological and antigenic processing of *V*. *cholerae* by host immune cells; specifically, that intestinal IgM and IgA anti-OSP antibody levels may have been so prominent within 10 days of vaccination and bound with such high avidity to intra-luminal wild-type *V*. *cholerae* organisms that not only was clinical protection present, but *V*. *cholerae* organisms were also not presented to human immune cells in Peyer’s patches or regional lymph nodes at an appreciable level. However, when experimental challenge was delayed for 90 days after vaccination, we did indeed find boosting of IgM and IgA responses targeting OSP, and new development of IgG responses targeting *V*. *cholerae* OSP. These observations might suggest that mucosal immune responses were then less prominent at this 90 day mark, and that this decrease allowed additional immunological processing and handling of *V*. *cholerae* organisms. Such a scenario could result in the observed rapid boosting of the IgM and IgA anti-OSP responses following the 90 day challenge to values that were actually higher than following primary immunization. This de facto boosting also appears to have led to isotype switching of the antibody response with development of IgG isotype antibodies targeting OSP. Whether there was antibody maturation and increased affinity afforded by this boosting was not assessed in this study. These observations agree with a prior analysis performed in North American volunteers who received a previous version of CVD 103-HgR and were then challenged with wild type *V*. *cholerae* O1 either 7, 30, or 180 days after vaccination [30]. In this previous analysis, antibody secreting cell (ASC) responses—a marker of mucosal immune responses [30]–were assessed following challenge. The earlier the challenge occurred, the more blunted was the ASC immune response [30]. Such data might suggest that repetitive exposure may result in consolidation of long-term immune responses and long-term protection against cholera in endemic zones. Such data might also suggest that a longer duration between vaccination with other oral cholera vaccines that are currently administered as two doses 14 days apart might be beneficial.

Although the mechanism has not been clearly elucidated, humans with blood group O are at increased risk of severe cholera, especially in its severest form: *cholera gravis* [31, 32]. In our current study, we found no difference in immune responses to *V*. *cholerae* OSP by A/B/O blood group. As such, CVD 103-HgR appears to be equivalently immunogenic with regard both to *V*. *cholerae* OSP as well as vibriocidal responses irrelevant of this major blood group category. These data suggest that the association of blood group and severity may not be directly due to differences in the ability to mount anti-OSP antibody responses.

We also assessed the ability of CVD 103-HgR to induce anti-OSP responses against *V*. *cholerae* Ogawa OSP, as well as Inaba OSP. *V*. *cholerae* can be classified into over 200 serogroups with *V*. *cholerae* O1 and O139 being able to cause epidemic cholera. *V*. *cholerae* O1 itself can be classified into a number of serotypes including Inaba and Ogawa, with the difference being the presence of a 2-*O*-methyl group in the non-reducing terminal sugar of the Ogawa OSP, which is absent from Inaba OSP [11]. Anti-Inaba and anti-Ogawa immune responses are largely cross-reactive and cross-protective, although it is thought that immune responses targeting *V*. *cholerae* O1 Inaba provide more protection against Ogawa than previous Ogawa infection provides against Inaba [11, 33, 34]. CVD 103-HgR is derived from an Inaba *V*. *cholerae* O1 serotype organism. We found that vaccination with CVD 103-HgR induced immune responses against both Inaba and Ogawa *V*. *cholerae* OSP, with responses being most prominent to the Inaba serotype. Our data suggest that CVD 103-HgR would provide protection against both Inaba and Ogawa serotype O1 organisms.

Our study has a number of limitations. We did not directly assess induction of mucosal immune responses targeting *V*. *cholerae* OSP, nor did we assess antibody affinity or avidity. We also did not assess duration of immune responses via long-lived plasma cells or memory B cell responses targeting *V*. *cholerae* OSP. This volunteer study also did not include children, who may respond differently to vaccination with CVD 103-HgR, especially to T cell-independent antigens such as OSP, although the induction of IgM responses to OSP as occurred in adults in this study would presumably still occur. Our study is only one of correlations, and is not able to assess the possible mechanism of protection that may be afforded by antibodies targeting *V*. *cholerae* OSP. Despite these limitations, our analysis shows that CVD 103-HgR induces immune responses targeting *V*. *cholerae* OSP; these responses are likely largely mucosal in nature; mucosal immune responses appear to be very high level soon after vaccination; and boosting of anti-OSP immune responses can occur following repetitive exposure to *V*. *cholerae*. We also found that blood group did not appear to affect the ability to mount an anti-OSP immune response, and that immune responses induced by CVD 103-HgR included antibodies targeting both Inaba and Ogawa serotype *V*. *cholerae*. Most importantly, we found that anti-OSP immune responses correlated with protection against diarrhea following wild-type experimental challenge, especially moderate or severe diarrhea. These data further support the growing body of evidence that immune responses targeting *V*. *cholerae* OSP may be a critical component of providing protection against cholera. Establishing the mechanism of this protection is warranted.

## Supporting information

S1 Table Serum Inaba OSP-specific antibody isotype fold increase (≥1.5) and Inaba-specific vibriocidal fold increase (≥4) on day 10 following vaccination as a predictor of protection against development of moderate/severe cholera following subsequent challenge; by challenge day subgroup (day 10 and day 90). (PDF)

S2 Table Serum Inaba OSP-specific antibody isotype fold increase (≥1.5) and Inaba-specific vibriocidal fold increase (≥4) on day 10 following vaccination as a predictor of protection against development of mild/moderate/severe cholera (any diarrhea) following subsequent challenge; by challenge day subgroup (day 10 and day 90). (PDF)

S1 Fig Antibody responses 10 days after vaccination with CVD 103-HgR by O blood group status of vaccine recipient. Serum IgM, IgA and IgG antibody responses targeting *V*. *cholerae* O1 Inaba and Ogawa OSP 10 days after oral vaccination with CVD 103-HgR by O blood group status of vaccine recipient. X axis indicates the time points of samples while Y-axis denotes OSP specific antibody responses. Each single dot indicates an individual OSP antibody value, horizontal bars indicate the geometric mean (GM), and error bars indicate 95% confidence intervals. Responders were defined as having an increase in kinetic ELISA units ≥ 1.5-fold on day 10 post-vaccination compared with day 0 pre-vaccination.
(PDF)

S2 Fig Antibody responses against OSP 10 days post vaccination in mild or moderate or severe versus no diarrhea. Serum IgM, IgA and IgG antibody responses targeting *V*. *cholerae* O1 Inaba and Ogawa OSP 10 days after oral vaccination with CVD 103-HgR and subsequent development of mild (<3L), moderate (≥3L) or severe (≥5L) diarrhea versus no diarrhea following wild type *V*. *cholerae* O1 Inaba experimental infection 10 or 90 days after vaccination. X axis indicates the time points of samples while Y-axis denotes OSP specific antibody responses. Each single dot indicates an individual OSP antibody value, horizontal bars indicate the geometric mean (GM), and error bars indicate 95% confidence intervals. Responders were defined as having an increase in kinetic ELISA units ≥ 1.5-fold on day 10 post-vaccination compared with day 0 pre-vaccination. Ina indicates Inaba and Oga indicates Ogawa.
(PDF)

S3 Fig Antibody responses against OSP 10 days post vaccination in moderate or severe versus no or mild diarrhea. Serum IgM, IgA and IgG antibody responses targeting *V*. *cholerae* O1 Inaba and Ogawa OSP 10 days after oral vaccination with CVD 103-HgR and subsequent development of moderate (≥3 − <5L) or severe (≥5L) diarrhea versus no or mild (<3L) diarrhea following wild type *V*. *cholerae* O1 Inaba experimental infection 10 or 90 days after vaccination. X axis indicates the time points of samples while Y-axis denotes OSP-specific antibody responses. Each single dot indicates an individual OSP antibody value, horizontal bars indicate the geometric mean (GM), and error bars indicate 95% confidence intervals. Responders were defined as having an increase in kinetic ELISA units ≥ 1.5-fold on day 10 post-vaccination compared with day 0 pre-vaccination. Ina indicates Inaba and Oga indicates Ogawa.
(PDF)

S4 Fig Serum vibriocidal antibody responses to *V*. *cholerae* O1 Inaba and Ogawa at different time points post-vaccination. Figure only contains results for samples collected prior to wild type experimental *V*. *cholerae* challenge. Day 0 is pre-vaccination. In total, 46 vaccinees are included until day 10, then 20 vaccinees until day 90. Each single dot indicates an individual vibriocidal antibody titer, horizontal bars indicate the geometric mean (GM) and error bars indicate 95% confidence intervals (CI). *P* values represent significant differences of the mean between groups. Asterisks denote significance between responder and non-responder frequency at every time point with baseline (*** *P* < 0.0001). Responders were defined as having a ≥4-fold increase in vibriocidal titer compared to the baseline (day 0).
(PDF)

S5 Fig Vibriocidal responses 10 days after oral vaccination with CVD 103-HgR by O blood group status of vaccine recipient. Each single dot represents an individual vibriocidal antibody titer, horizontal bars represent the geometric mean (GM) and error bars represent 95% confidence intervals (CI). Responders were defined as having a ≥ 4-fold increase in vibriocidal value on day 10 post-vaccination compared with day 0 pre-vaccination.
(PDF)

S6 Fig Serum vibriocidal antibody responses to Inaba and Ogawa in vaccinees of day 10 challenged group. Serum vibriocidal antibody responses to *V*. *cholerae* O1 Inaba and Ogawa at different time points post-vaccination in recipients (N = 26) of oral cholera vaccine CVD 103-HgR who were then experimentally challenged with wild type *V*. *cholerae* O1 Inaba N16961 10 days after vaccination. Day 0 is pre-vaccination. Other dates are timed from vaccination. Each single dot denotes an individual vibriocidal antibody titer, horizontal bars denote the geometric mean (GM) and error bars denote 95% confidence intervals (CI). *P* values represent significant differences of the mean between groups. Asterisks denote significance between responder and non-responder frequency of every time point compared with baseline (*** *P* < 0.0001). Responders were defined as having a ≥4-fold increase in vibriocidal titer compared to the baseline (day 0).
(PDF)

S7 Fig Serum vibriocidal antibody responses to Inaba and Ogawa in vaccinees of day 90 challenged group. Serum vibriocidal antibody responses to *V*. *cholerae* O1 Inaba and Ogawa at different time points post-vaccination in recipients (N = 20) of oral cholera vaccine CVD 103-HgR who were then experimentally challenged with wild type *V*. *cholerae* O1 Inaba N16961 90 days after vaccination. Day 0 is pre-vaccination. Other dates are timed from vaccination. Each single dot represents an individual vibriocidal antibody titer, horizontal bars represent the geometric mean (GM) and error bars represent 95% confidence intervals (CI). *P* values represent significant differences of the mean between groups. Asterisks denote significance between responder and non-responder frequency of every time point compared with baseline (*** *P* < 0.0001 and ** *P* < 0.01). Responders were defined as having a ≥4-fold increase in vibriocidal titer compared to the baseline (day 0).
(PDF)

S8 Fig Serum vibriocidal antibody responses 10 days post vaccination in moderate or severe versus no diarrhea. Serum vibriocidal antibody responses to *V*. *cholerae* O1 Inaba and Ogawa 10 days post-vaccination and subsequent development of moderate (≥3L) or severe (≥5L) diarrhea versus no diarrhea following wild type *V*. *cholerae* O1 Inaba experimental infection 10 or 90 days after vaccination. Each single dot indicates an individual vibriocidal antibody titer, horizontal bars indicate the geometric mean (GM) and error bars indicate 95% confidence intervals (CI). Responders were defined as having ≥4-fold increase in vibriocidal value (day 10 post-vaccination compared to day 0 pre-vaccination). Asterisks denote significant difference (** *P* < 0.01) of responder frequency between no diarrhea versus moderate/severe diarrhea group in Fisher’s exact test.
(PDF)

S9 Fig Serum vibriocidal antibody responses 10 days post vaccination in mild or moderate or severe versus no diarrhea. Serum vibriocidal antibody responses to *V*. *cholerae* O1 Inaba and Ogawa 10 days post-vaccination and subsequent development of mild (<3L), moderate (≥3 − <5L) or severe (≥5L) diarrhea versus no diarrhea following wild type *V*. *cholerae* O1 Inaba experimental infection 10 or 90 days after vaccination. Each single dot denotes an individual vibriocidal antibody titer, horizontal bars denote the geometric mean (GM) and error bars denote 95% confidence intervals (CI). Responders were defined as having ≥4-fold increase in vibriocidal value (day 10 post-vaccination compared to day 0 pre-vaccination). Asterisks denote significant difference (* *P* < 0.05, ** *P* < 0.01) of responder frequency between no diarrhea versus mild/moderate/severe diarrhea group in Fisher’s exact test.
(PDF)

S10 Fig Serum vibriocidal antibody responses 10 days post vaccination in moderate or severe versus no or mild diarrhea. Serum vibriocidal antibody responses to *V*. *cholerae* O1 Inaba and Ogawa 10 days post-vaccination and subsequent development of mild (<3 L), moderate (≥3L) or severe (≥5L) diarrhea versus no diarrhea following wild type *V*. *cholerae* O1 Inaba experimental infection 10 or 90 days after vaccination. Each single dot represents an individual vibriocidal antibody titer, horizontal bars represent the geometric mean (GM) and error bars represent 95% confidence intervals (CI). Responders were defined as having ≥4-fold increase in vibriocidal value (day 10 post-vaccination compared to day 0 pre-vaccination). Asterisks indicates significant difference (** *P* < 0.01) of responder frequency between no/mild diarrhea versus moderate/severe diarrhea group in Fisher’s exact test.
(PDF)

S11 Fig Correlation of fold-increase (day 10 to day 0) of vibriocidal antibody responses against *V*. *cholerae* O1 Inaba and cumulative diarrhea following experimental challenge 10 or 90 days post vaccination. Dashed horizontal lines mark moderate (3L) or severe (5L) diarrheal and dashed vertical line denotes 4-fold vibriocidal change value (day 10 post vaccination compared to day 0 pre-vaccination).
(PDF)

S12 Fig Correlation of fold-increase (day 10 to day 0) of vibriocidal antibody responses against *V*. *cholerae* O1 Ogawa and cumulative diarrhea following experimental challenge 10 or 90 days post vaccination. Dashed horizontal lines mark moderate (3L) or severe (5L) diarrheal and dashed vertical line denotes 4-fold vibriocidal change value (day 10 post vaccination compared to day 0 pre-vaccination).
(PDF)

S13 Fig Correlation of serum vibriocidal antibody responses to *V*. *cholerae* O1 Inaba with IgM, IgA, and IgG responses to Inaba OSP 10 days after vaccination. (PDF)
