# Supplementary material for: Anti-O-specific polysaccharide (OSP) immune responses following vaccination with oral cholera vaccine CVD 103-HgR correlate with protection against cholera after infection with wild-type Vibrio cholerae O1 El Tor Inaba in North American volunteers
Source: PLoS Negl Trop Dis. 2018 Apr 6;12(4):e0006376. doi: 10.1371/journal.pntd.0006376 (PMC5906022; doi:10.1371/journal.pntd.0006376)
Supplement: S1 Table — (PDF) [file pntd.0006376.s001.pdf]

S1 Table. Serum Inaba OSP-specific antibody isotype fold increase (≥1.5) and Inaba-specific vibriocidal fold increase (≥4) on day 10 following vaccination as a predictor of protection against development of moderate/severe cholera following subsequent challenge; by challenge day subgroup (day 10 and day 90).

Inaba OSP-specific antibody isotype fold increase; and Inaba vibriocidal fold increase	Fold increase on day10 *	Moderate/ Severe diarrhea		*P* Value	Fold increase at day 10 in 10 day challenge group**	Moderate/ Severe diarrhea		*P* Value	Fold increase at day 10 in 90 day challenge group***	Moderate/ Severe diarrhea		*P* Value
		Yes	No			Yes	No			Yes	No	
Any of 3 isotypes or all three IgM, A, and G ≥1.5	Yes	0	27	0.01	Yes	0	18	0.09	Yes	0	9	0.22
	No	5	14		No	2	6		No	3	8	
Either or both IgM, A ≥1.5	Yes	0	27	0.01	Yes	0	18	0.09	Yes	0	9	0.22
	No	5	14		No	2	6		No	3	8	
Either or both IgM, G ≥1.5	Yes	0	26	0.01	Yes	0	17	0.11	Yes	0	9	0.22
	No	5	15		No	2	7		No	3	8	
Either or both IgA, G ≥1.5	Yes	0	19	0.07	Yes	0	12	0.48	Yes	0	7	0.52
	No	5	22		No	2	12		No	3	10	
IgM ≥1.5	Yes	0	26	0.01	Yes	0	17	0.11	Yes	0	9	0.22
	No	5	15		No	2	7		No	3	8	
IgA ≥1.5	Yes	0	19	0.07	Yes	0	12	0.48	Yes	0	7	0.52
	No	5	22		No	2	12		No	3	10	
IgG ≥1.5	Yes	0	3	1.00	Yes	0	2	1.00	Yes	0	1	1.00

	No	5	38		No	2	22		No	3	16	
Vibriocidal ≥ 4.0	Yes	2	39	0.01	Yes	1	23	0.15	Yes	1	16	0.05
	No	3	2		No	1	1		No	2	1	

*Represents increase on day 10 after vaccination from day 0 anti-OSP value; combining day 10 and day 90 challenge groups

** Day 10 fold increase after vaccination over day 0 for day 10 challenge group only

*** Day 10 fold increase after vaccination over day 0 for day 90 challenge group only
