# Supplementary material for: Anti-O-specific polysaccharide (OSP) immune responses following vaccination with oral cholera vaccine CVD 103-HgR correlate with protection against cholera after infection with wild-type Vibrio cholerae O1 El Tor Inaba in North American volunteers
Source: PLoS Negl Trop Dis. 2018 Apr 6;12(4):e0006376. doi: 10.1371/journal.pntd.0006376 (PMC5906022; doi:10.1371/journal.pntd.0006376)
Supplement: S2 Table — (PDF) [file pntd.0006376.s002.pdf]

S2 Table. Serum Inaba OSP-specific antibody isotype fold increase (≥1.5) and Inaba-specific vibriocidal fold increase (≥4) on day 10 following vaccination as a predictor of protection against development of mild/moderate/severe cholera (any diarrhea) following subsequent challenge; by challenge day subgroup (day 10 and day 90).

Inaba OSP specific antibody isotype fold increase; and Inaba vibriocidal fold increase	Fold increase on day10 *	Mild/ Moderate/ Severe Cholera		*P* Value	Fold increase at day 10 in 10 day challenge group**	Mild/ Moderate /Severe Cholera		*P* Value	Fold increase at day 10 in 90 day challenge group***	Mild/ Moderate/ Severe Cholera		*P* Value
		Yes	No			Yes	No			Yes	No	
Any of 3 or all IgM, A, G ≥1.5	Yes	6	21	0.03	Yes	2	16	0.28	Yes	4	5	0.36
	No	11	8		No	3	5		No	8	3	
Either or both IgM, A ≥1.5	Yes	6	21	0.03	Yes	2	16	0.28	Yes	4	5	0.36
	No	11	8		No	3	5		No	8	3	
Either or both IgM, G ≥1.5	Yes	6	20	0.04	Yes	2	16	0.28	Yes	4	5	0.36
	No	11	9		No	3	5		No	8	3	
Either or both IgA, G ≥1.5	Yes	3	16	0.02	Yes	1	11	0.33	Yes	2	5	0.06
	No	14	13		No	4	10		No	10	3	
IgM ≥1.5	Yes	6	20	0.04	Yes	2	15	0.28	Yes	4	5	0.36
	No	11	9		No	3	6		No	8	3	
IgA ≥1.5	Yes	3	16	0.02	Yes	1	11	0.33	Yes	2	5	0.06
	No	14	13		No	4	10		No	10	3	
IgG ≥1.5	Yes	0	3	0.29	Yes	0	2	1.00	Yes	0	1	0.40
	No	17	26		No	5	19		No	12	7	
Vibriocidal ≥ 4.0	Yes	13	28	0.06	Yes	4	20	0.35	Yes	9	8	0.24
	No	4	1		No	1	1		No	3	0	

*Represents day 10 fold increase after vaccination from day 0 anti-OSP value; combining day 10 and day 90 challenge groups

** Day 10 after vaccination fold increase over day 0 for day 10 challenge group only

*** Day 10 after vaccination fold increase over day 0 for day 90 challenge group only
