# Supplementary figures and images for: Anti-O-specific polysaccharide (OSP) immune responses following vaccination with oral cholera vaccine CVD 103-HgR correlate with protection against cholera after infection with wild-type Vibrio cholerae O1 El Tor Inaba in North American volunteers
Source: PLoS Negl Trop Dis. 2018 Apr 6;12(4):e0006376. doi: 10.1371/journal.pntd.0006376 (PMC5906022; doi:10.1371/journal.pntd.0006376)

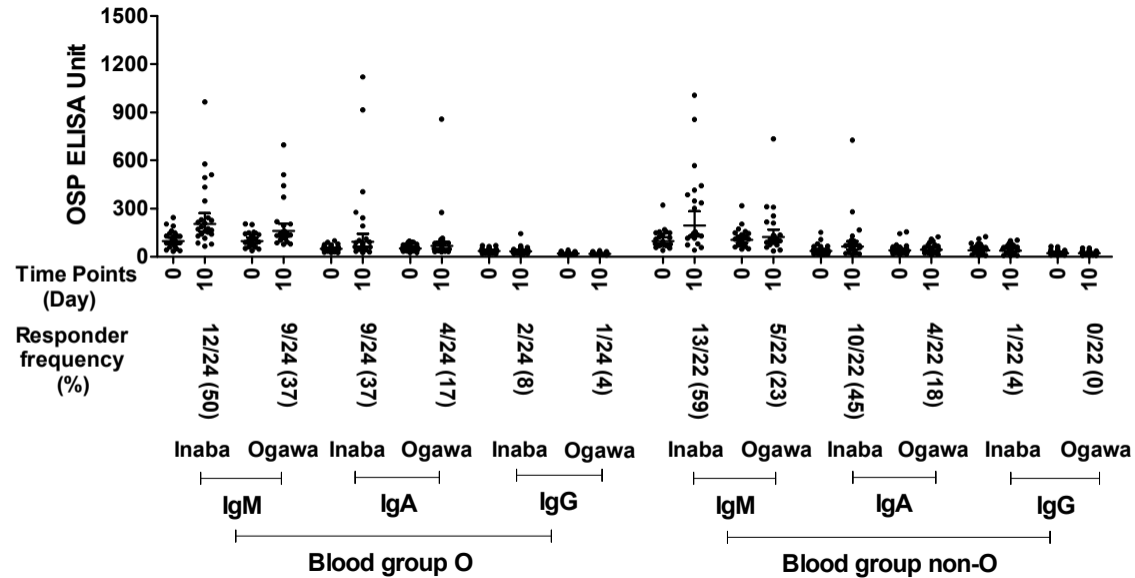

Supplement: S1 Fig — Serum IgM, IgA and IgG antibody responses targeting V. cholerae O1 Inaba and Ogawa OSP 10 days after oral vaccination with CVD 103-HgR by O blood group status of vaccine recipient. X axis indicates the time points of samples while Y-axis denotes OSP specific antibody responses. Each single dot indicates an individual OSP antibody value, horizontal bars indicate the geometric mean (GM), and error bars indicate 95% confidence intervals. Responders were defined as having an increase in kinetic ELISA units ≥ 1.5-fold on day 10 post-vaccination compared with day 0 pre-vaccination. (PDF) [file pntd.0006376.s003.pdf]

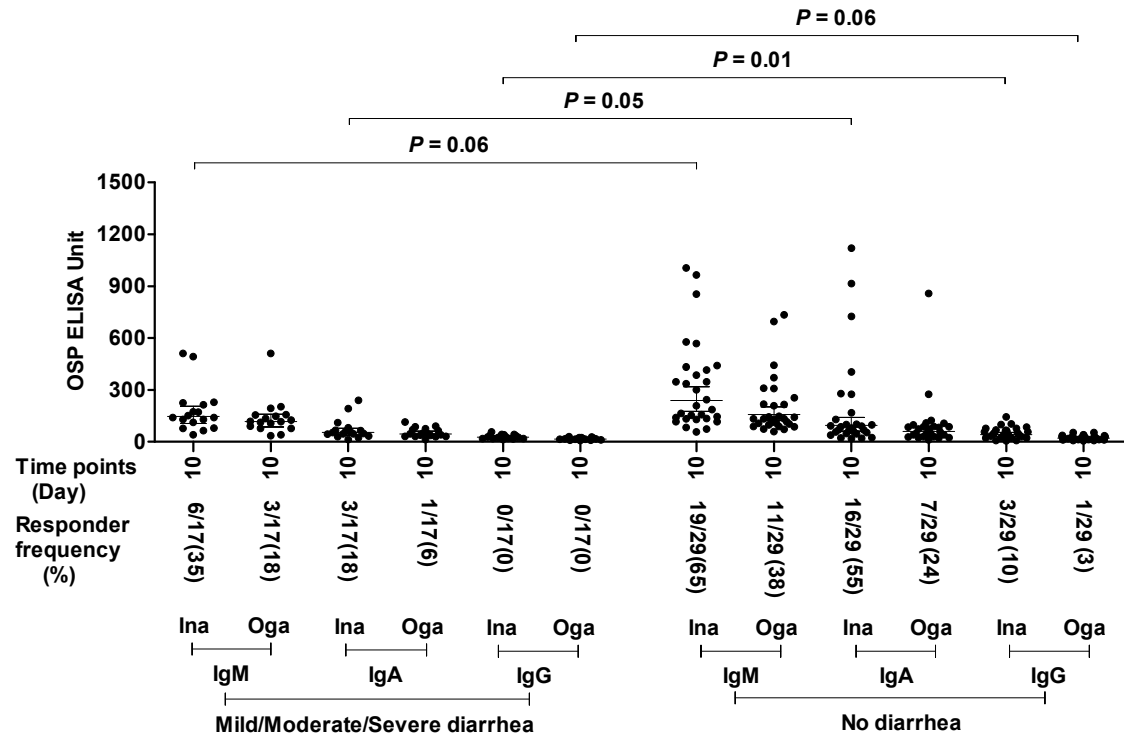

Supplement: S2 Fig — Serum IgM, IgA and IgG antibody responses targeting V. cholerae O1 Inaba and Ogawa OSP 10 days after oral vaccination with CVD 103-HgR and subsequent development of mild (<3L), moderate (≥3L) or severe (≥5L) diarrhea versus no diarrhea following wild type V. cholerae O1 Inaba experimental infection 10 or 90 days after vaccination. X axis indicates the time points of samples while Y-axis denotes OSP specific antibody responses. Each single dot indicates an individual OSP antibody value, horizontal bars indicate the geometric mean (GM), and error bars indicate 95% confidence intervals. Responders were defined as having an increase in kinetic ELISA units ≥ 1.5-fold on day 10 post-vaccination compared with day 0 pre-vaccination. Ina indicates Inaba and Oga indicates Ogawa. (PDF) [file pntd.0006376.s004.pdf]

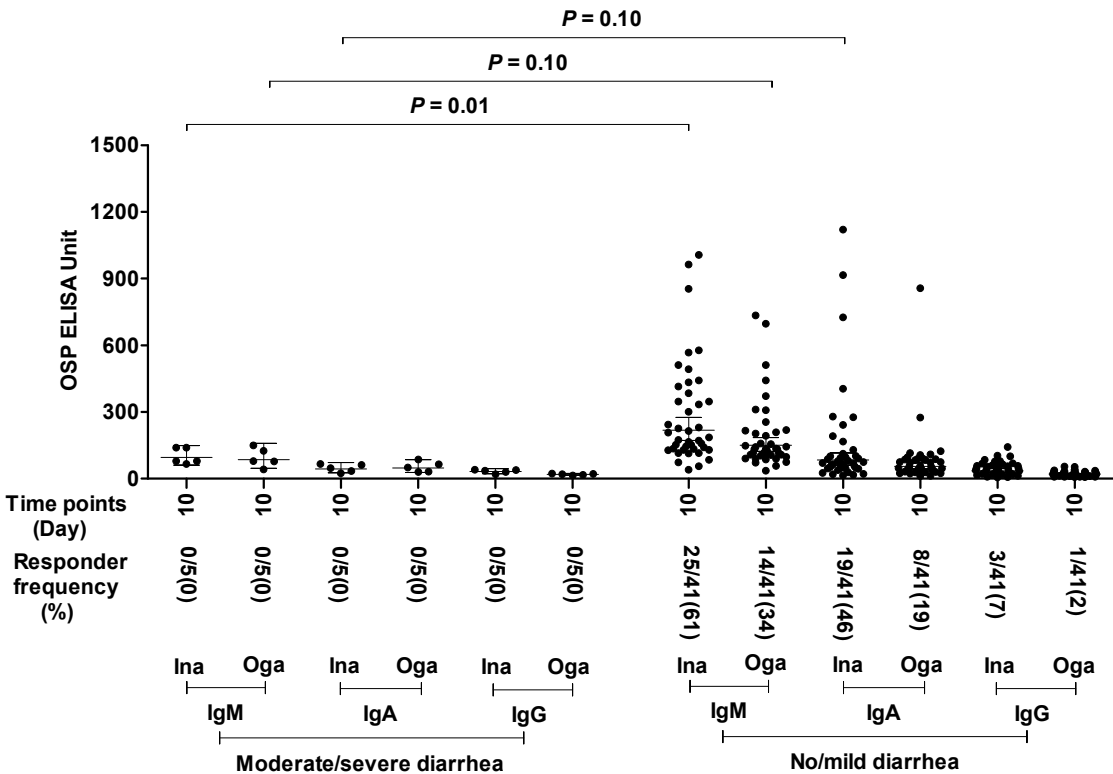

Supplement: S3 Fig — Serum IgM, IgA and IgG antibody responses targeting V. cholerae O1 Inaba and Ogawa OSP 10 days after oral vaccination with CVD 103-HgR and subsequent development of moderate (≥3 − <5L) or severe (≥5L) diarrhea versus no or mild (<3L) diarrhea following wild type V. cholerae O1 Inaba experimental infection 10 or 90 days after vaccination. X axis indicates the time points of samples while Y-axis denotes OSP-specific antibody responses. Each single dot indicates an individual OSP antibody value, horizontal bars indicate the geometric mean (GM), and error bars indicate 95% confidence intervals. Responders were defined as having an increase in kinetic ELISA units ≥ 1.5-fold on day 10 post-vaccination compared with day 0 pre-vaccination. Ina indicates Inaba and Oga indicates Ogawa. (PDF) [file pntd.0006376.s005.pdf]

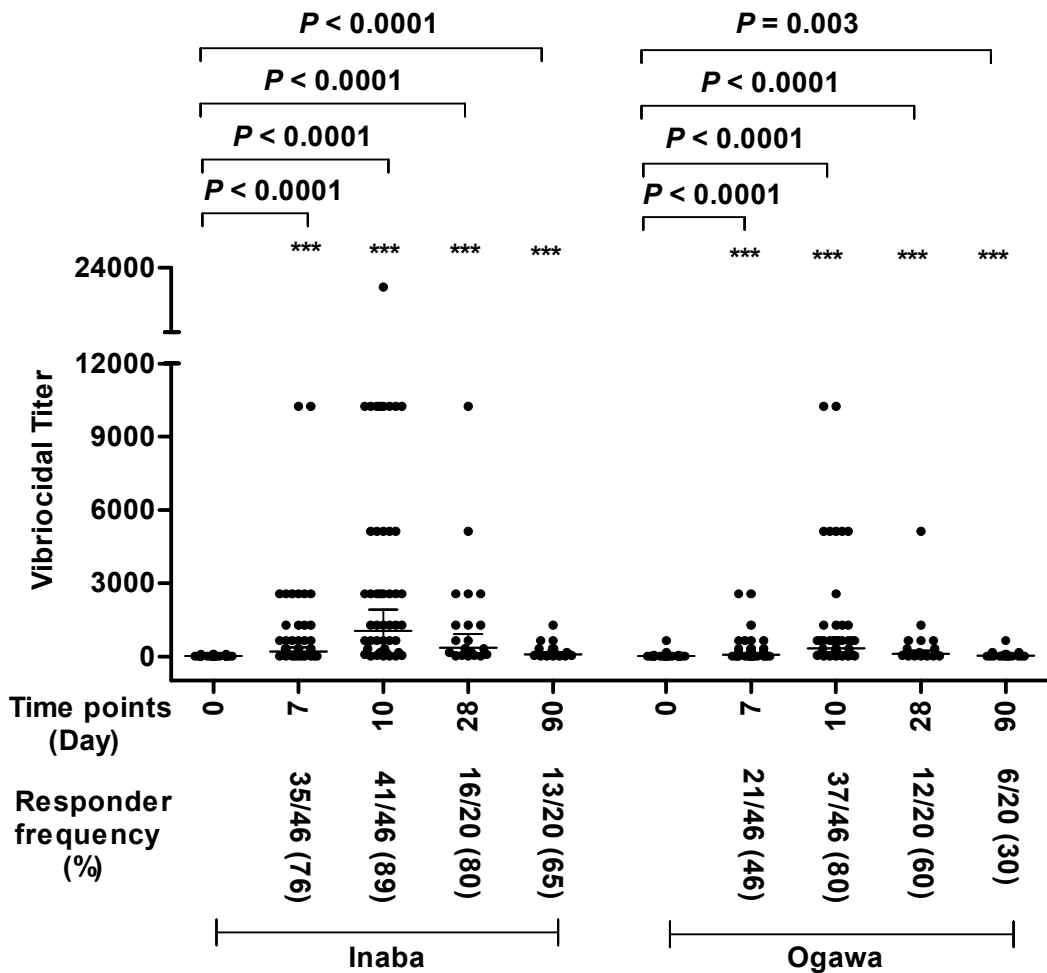

Supplement: S4 Fig — Figure only contains results for samples collected prior to wild type experimental V. cholerae challenge. Day 0 is pre-vaccination. In total, 46 vaccinees are included until day 10, then 20 vaccinees until day 90. Each single dot indicates an individual vibriocidal antibody titer, horizontal bars indicate the geometric mean (GM) and error bars indicate 95% confidence intervals (CI). P values represent significant differences of the mean between groups. Asterisks denote significance between responder and non-responder frequency at every time point with baseline (*** P < 0.0001). Responders were defined as having a ≥4-fold increase in vibriocidal titer compared to the baseline (day 0). (PDF) [file pntd.0006376.s006.pdf]

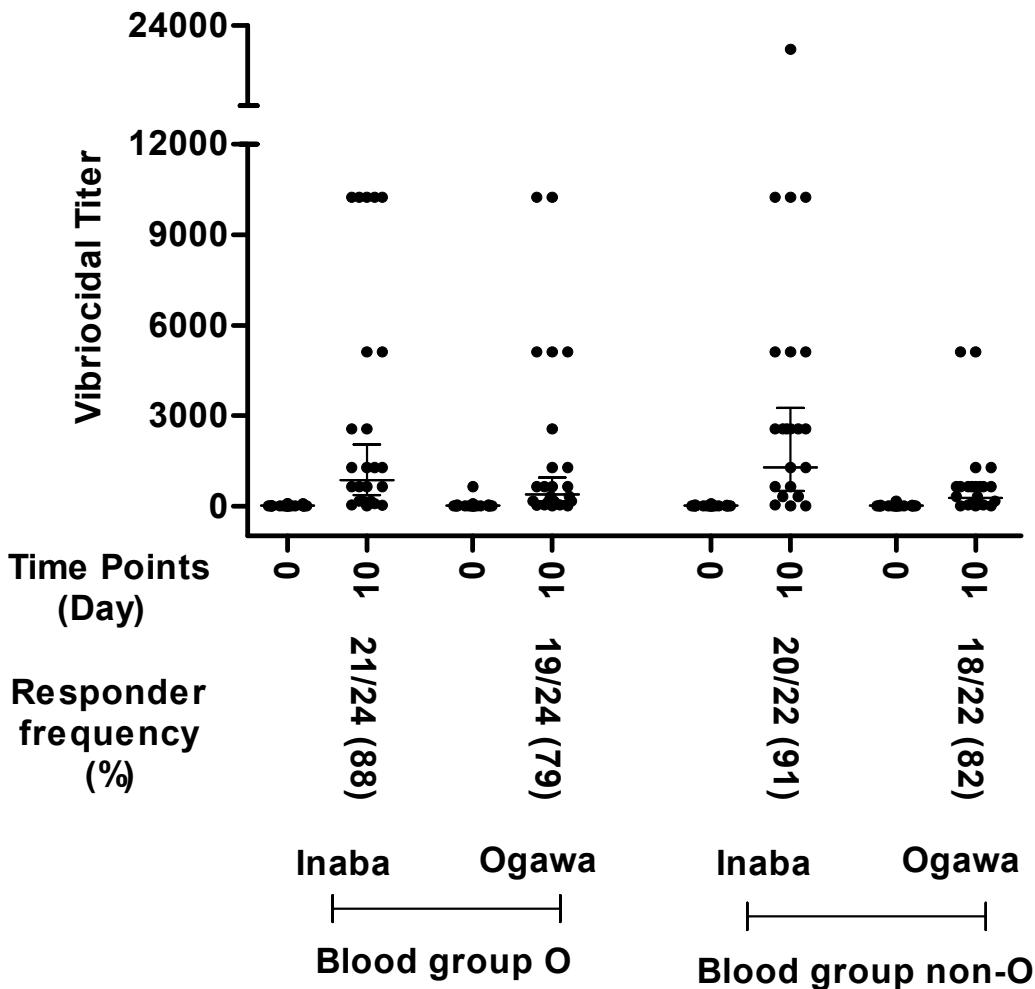

Supplement: S5 Fig — Each single dot represents an individual vibriocidal antibody titer, horizontal bars represent the geometric mean (GM) and error bars represent 95% confidence intervals (CI). Responders were defined as having a ≥ 4-fold increase in vibriocidal value on day 10 post-vaccination compared with day 0 pre-vaccination. (PDF) [file pntd.0006376.s007.pdf]

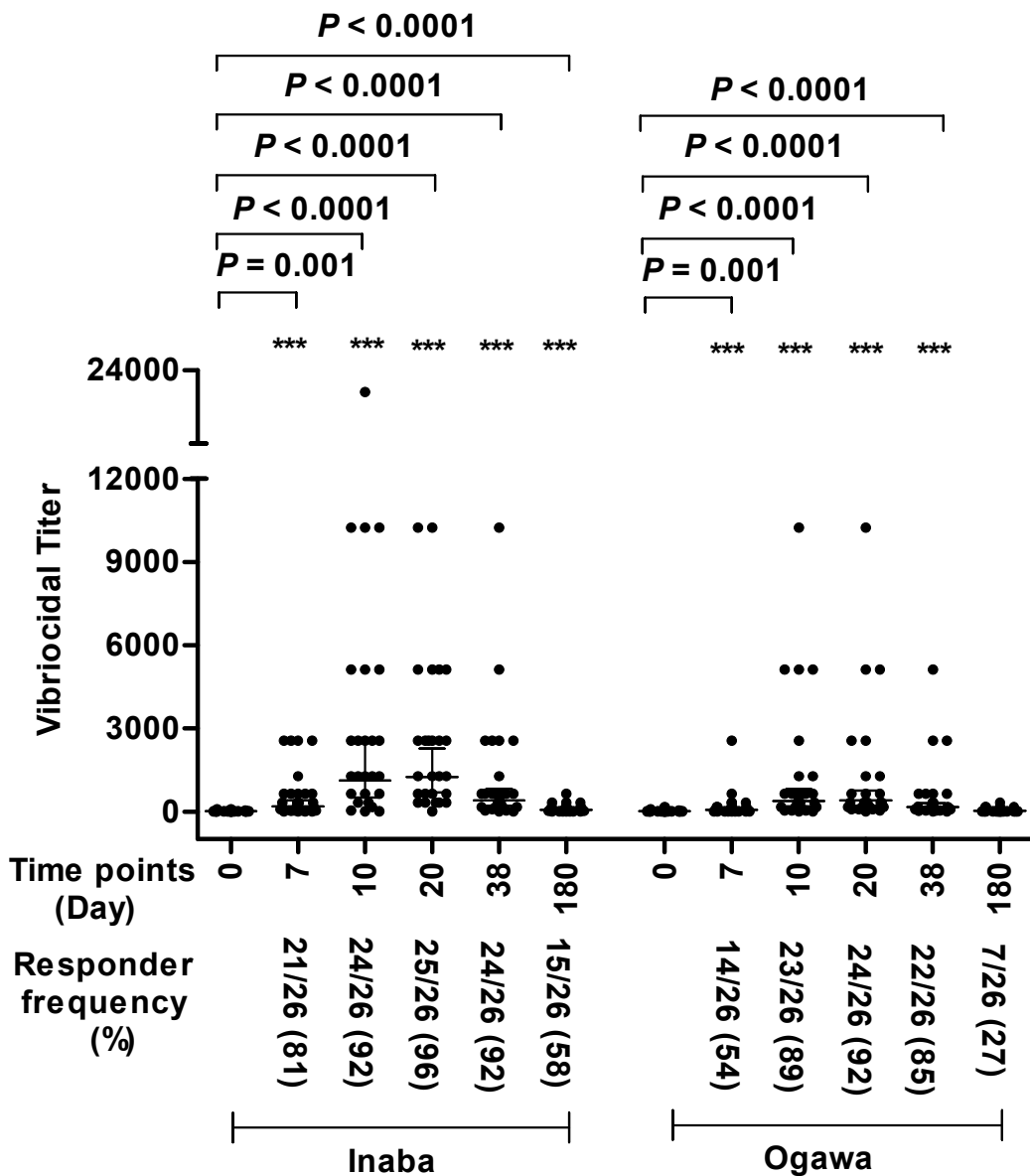

Supplement: S6 Fig — Serum vibriocidal antibody responses to V. cholerae O1 Inaba and Ogawa at different time points post-vaccination in recipients (N = 26) of oral cholera vaccine CVD 103-HgR who were then experimentally challenged with wild type V. cholerae O1 Inaba N16961 10 days after vaccination. Day 0 is pre-vaccination. Other dates are timed from vaccination. Each single dot denotes an individual vibriocidal antibody titer, horizontal bars denote the geometric mean (GM) and error bars denote 95% confidence intervals (CI). P values represent significant differences of the mean between groups. Asterisks denote significance between responder and non-responder frequency of every time point compared with baseline (*** P < 0.0001). Responders were defined as having a ≥4-fold increase in vibriocidal titer compared to the baseline (day 0). (PDF) [file pntd.0006376.s008.pdf]

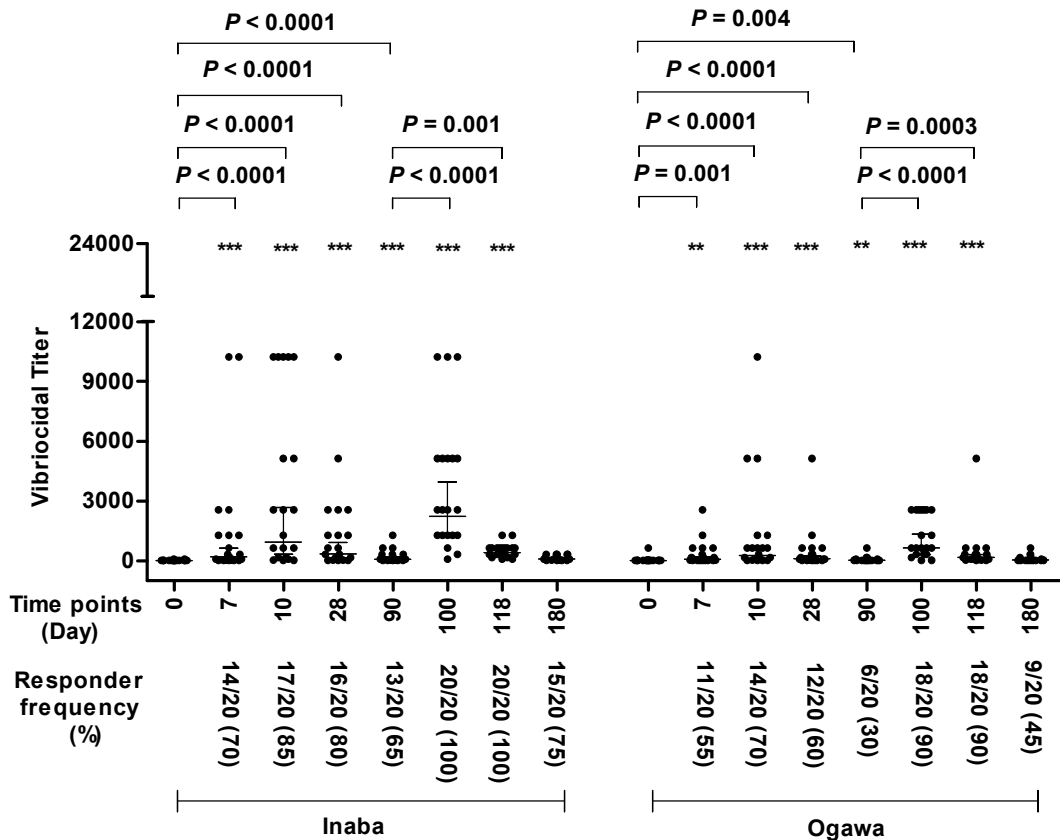

Supplement: S7 Fig — Serum vibriocidal antibody responses to V. cholerae O1 Inaba and Ogawa at different time points post-vaccination in recipients (N = 20) of oral cholera vaccine CVD 103-HgR who were then experimentally challenged with wild type V. cholerae O1 Inaba N16961 90 days after vaccination. Day 0 is pre-vaccination. Other dates are timed from vaccination. Each single dot represents an individual vibriocidal antibody titer, horizontal bars represent the geometric mean (GM) and error bars represent 95% confidence intervals (CI). P values represent significant differences of the mean between groups. Asterisks denote significance between responder and non-responder frequency of every time point compared with baseline (*** P < 0.0001 and ** P < 0.01). Responders were defined as having a ≥4-fold increase in vibriocidal titer compared to the baseline (day 0). (PDF) [file pntd.0006376.s009.pdf]

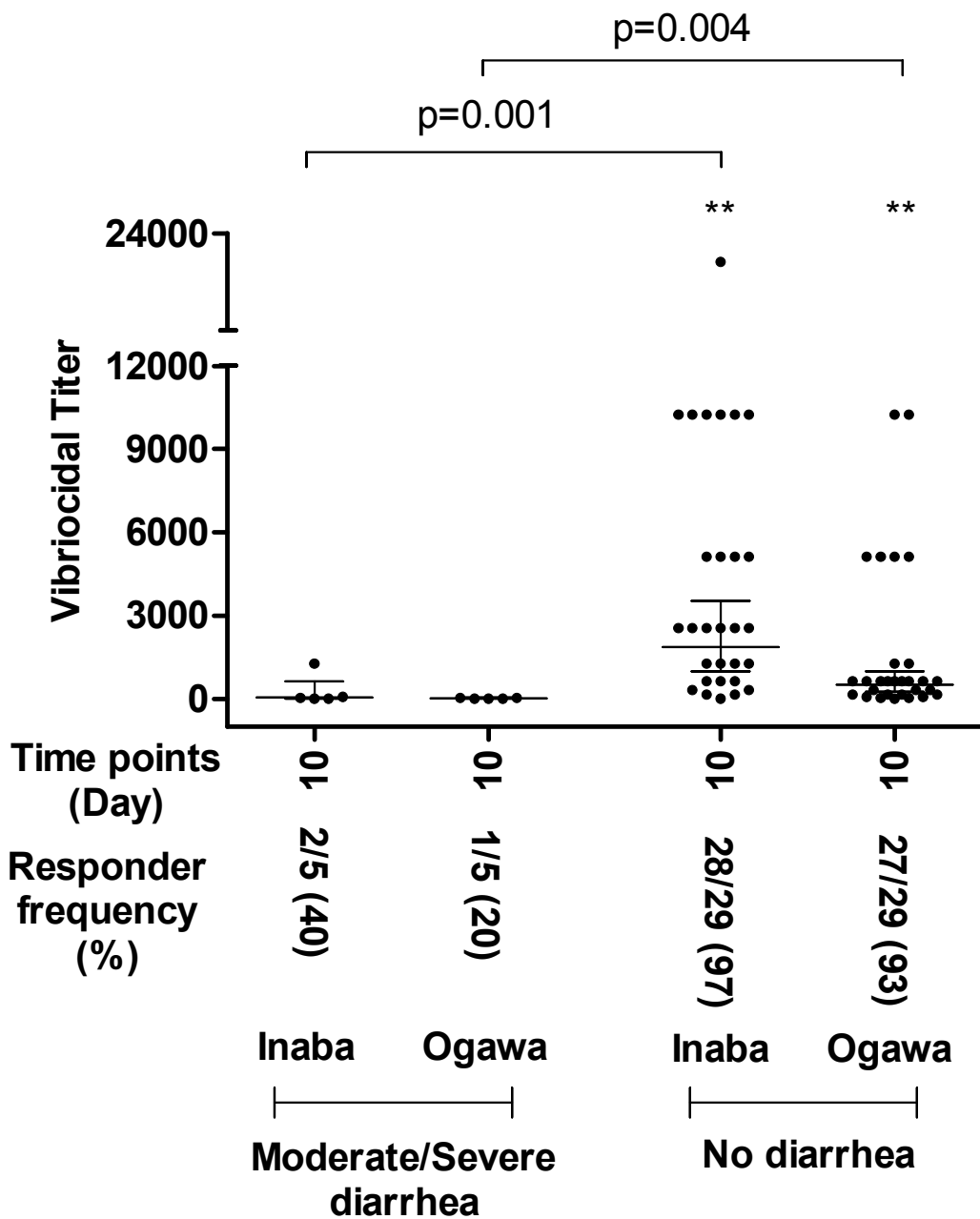

Supplement: S8 Fig — Serum vibriocidal antibody responses to V. cholerae O1 Inaba and Ogawa 10 days post-vaccination and subsequent development of moderate (≥3L) or severe (≥5L) diarrhea versus no diarrhea following wild type V. cholerae O1 Inaba experimental infection 10 or 90 days after vaccination. Each single dot indicates an individual vibriocidal antibody titer, horizontal bars indicate the geometric mean (GM) and error bars indicate 95% confidence intervals (CI). Responders were defined as having ≥4-fold increase in vibriocidal value (day 10 post-vaccination compared to day 0 pre-vaccination). Asterisks denote significant difference (** P < 0.01) of responder frequency between no diarrhea versus moderate/severe diarrhea group in Fisher’s exact test. (PDF) [file pntd.0006376.s010.pdf]

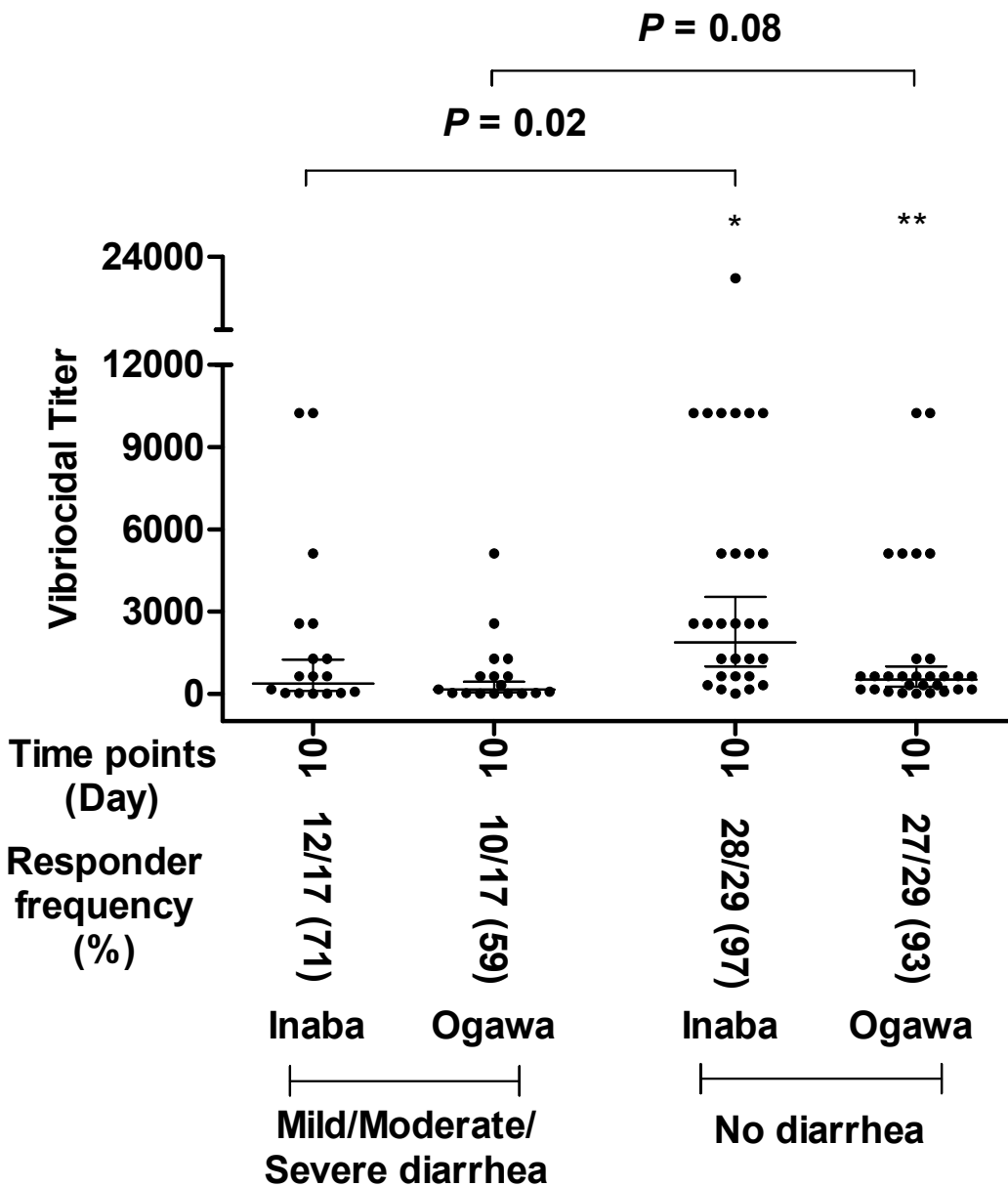

Supplement: S9 Fig — Serum vibriocidal antibody responses to V. cholerae O1 Inaba and Ogawa 10 days post-vaccination and subsequent development of mild (<3L), moderate (≥3 − <5L) or severe (≥5L) diarrhea versus no diarrhea following wild type V. cholerae O1 Inaba experimental infection 10 or 90 days after vaccination. Each single dot denotes an individual vibriocidal antibody titer, horizontal bars denote the geometric mean (GM) and error bars denote 95% confidence intervals (CI). Responders were defined as having ≥4-fold increase in vibriocidal value (day 10 post-vaccination compared to day 0 pre-vaccination). Asterisks denote significant difference (* P < 0.05, ** P < 0.01) of responder frequency between no diarrhea versus mild/moderate/severe diarrhea group in Fisher’s exact test. (PDF) [file pntd.0006376.s011.pdf]

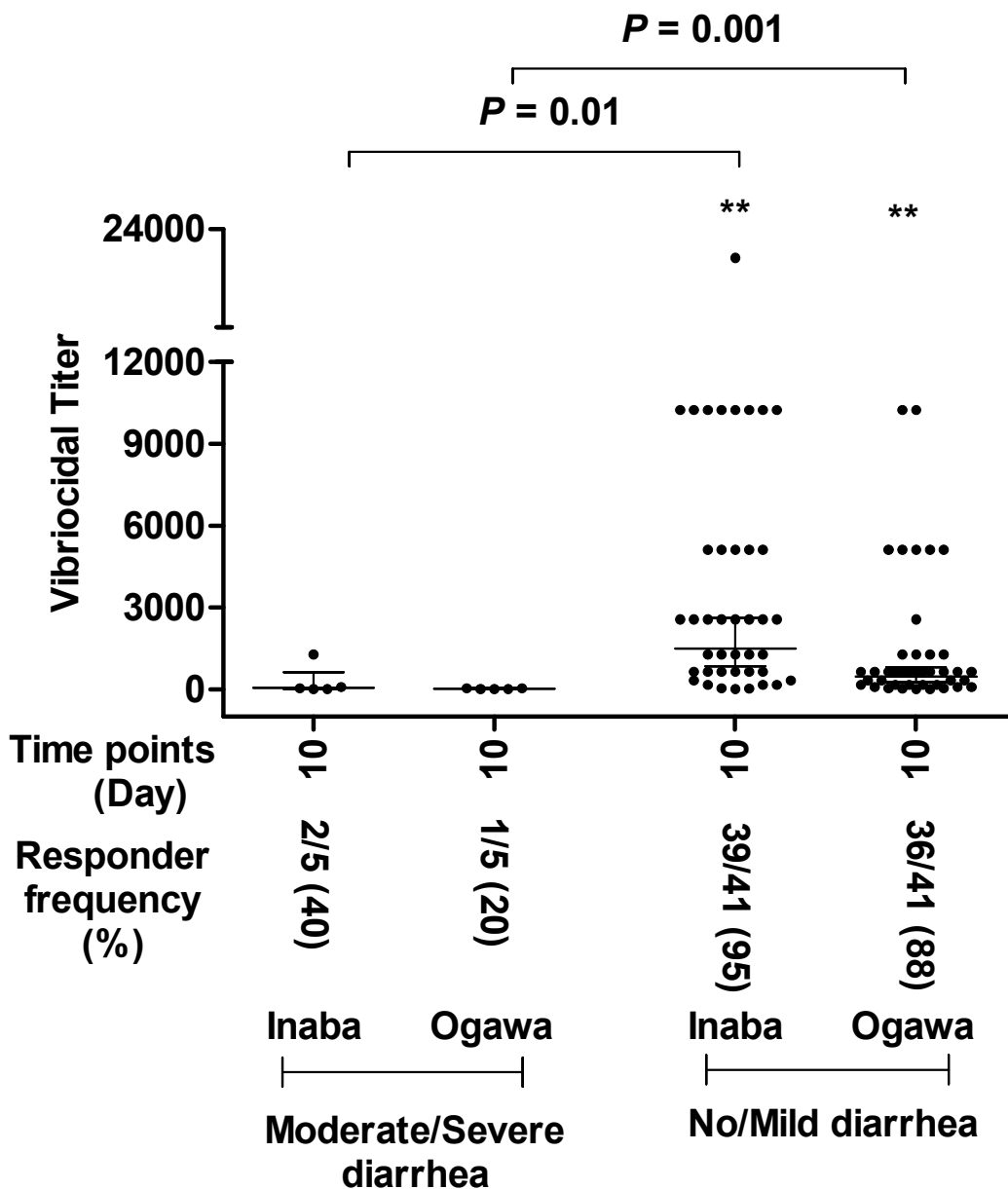

Supplement: S10 Fig — Serum vibriocidal antibody responses to V. cholerae O1 Inaba and Ogawa 10 days post-vaccination and subsequent development of mild (<3 L), moderate (≥3L) or severe (≥5L) diarrhea versus no diarrhea following wild type V. cholerae O1 Inaba experimental infection 10 or 90 days after vaccination. Each single dot represents an individual vibriocidal antibody titer, horizontal bars represent the geometric mean (GM) and error bars represent 95% confidence intervals (CI). Responders were defined as having ≥4-fold increase in vibriocidal value (day 10 post-vaccination compared to day 0 pre-vaccination). Asterisks indicates significant difference (** P < 0.01) of responder frequency between no/mild diarrhea versus moderate/severe diarrhea group in Fisher’s exact test. (PDF) [file pntd.0006376.s012.pdf]

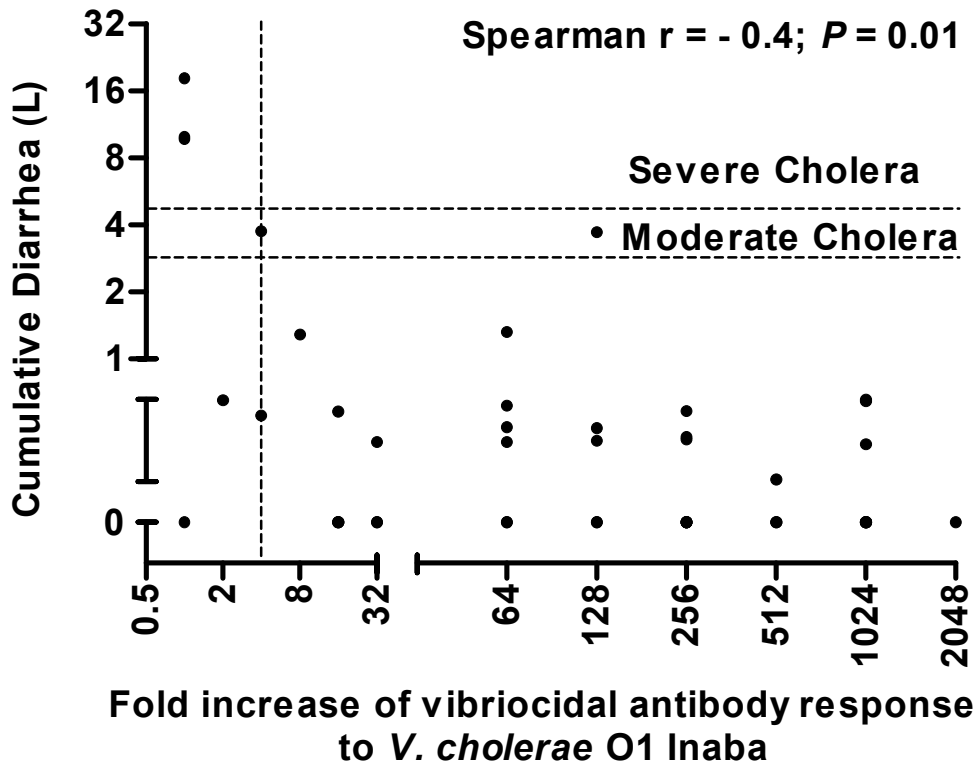

Supplement: S11 Fig — Dashed horizontal lines mark moderate (3L) or severe (5L) diarrheal and dashed vertical line denotes 4-fold vibriocidal change value (day 10 post vaccination compared to day 0 pre-vaccination). (PDF) [file pntd.0006376.s013.pdf]

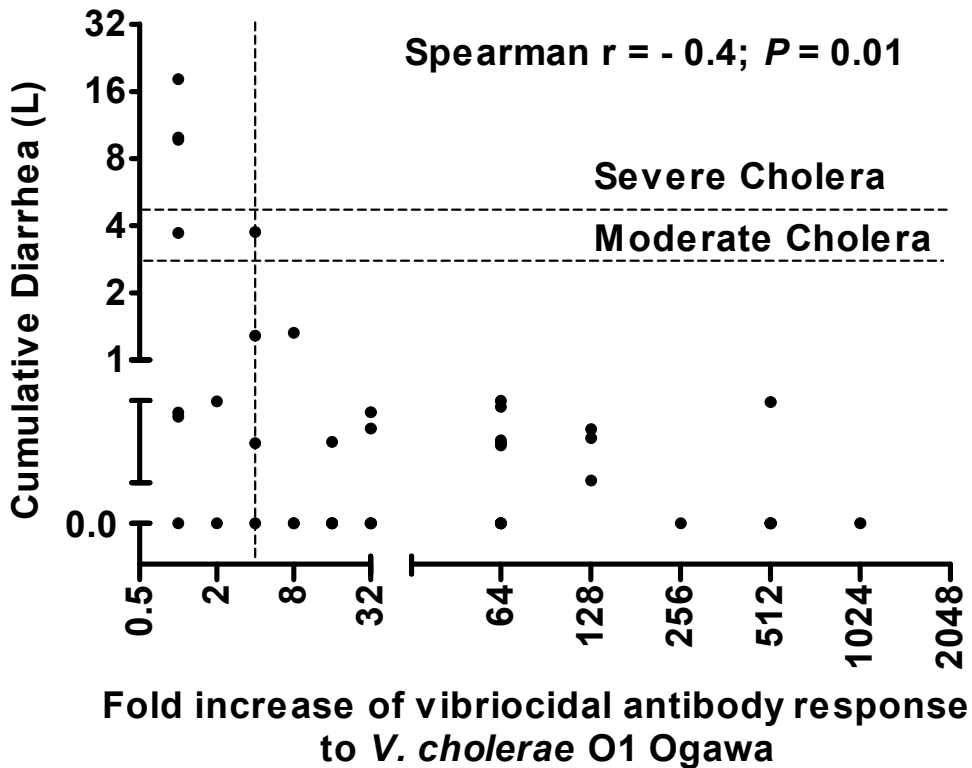

Supplement: S12 Fig — Dashed horizontal lines mark moderate (3L) or severe (5L) diarrheal and dashed vertical line denotes 4-fold vibriocidal change value (day 10 post vaccination compared to day 0 pre-vaccination). (PDF) [file pntd.0006376.s014.pdf]

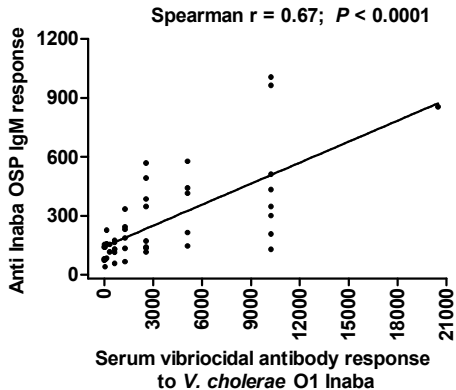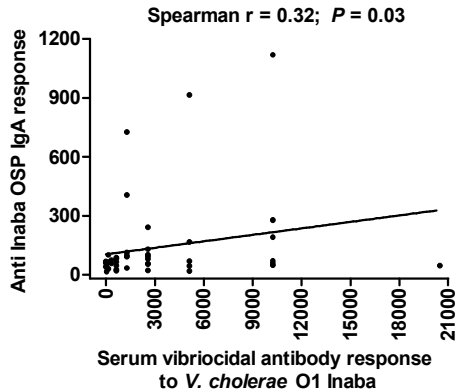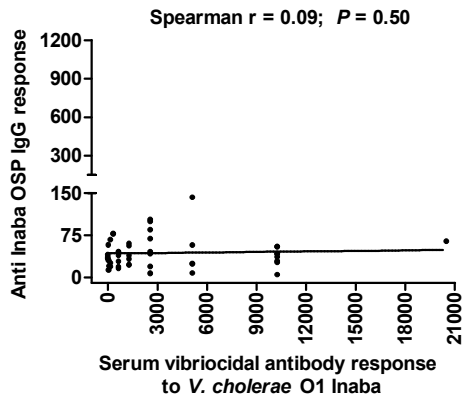

Supplement: S13 Fig — (PDF) [file pntd.0006376.s015.pdf]
